# Monounsaturated fatty acid biosynthesis is critical for streptococcal envelope homeostasis and stress tolerance

**DOI:** 10.1128/jb.00026-26

**Published:** 2026-08-07

**Authors:** Jonathon L. Baker, Jonah Tang, Mingzhe Guo, Felipe Fabrício Farias-da-Silva, Matthew Barbisan, Molly Burnside, Kaitlin Crofton, Samantha Williams, Saarin Rao, Michelle Lee, Sadie G. Drucker, Daniel Kim, Dustin Higashi, Justin Merritt, Yujiro Hirose, Jan-Willem Veening, Victor Nizet

**Affiliations:** 1 Department of Biomaterial & Biomedical Sciences, OHSU School of Dentistry 212162 , Portland, Oregon, USA; 2 Department of Biomedical Engineering, OHSU School of Medicine 89020 , Portland, Oregon, USA; 3 KCGIP, Knight Campus for Accelerating Scientific Impact, University of Oregon 3265, Eugene, Oregon, USA; 4 Department of Public Health, Pediatric Dentistry and Orthodontics, Faculdade de Odontologia de Piracicaba Universidade Estadual de Campinas 245124, Piracicaba, São Paulo, Brazil; 5 Department of Biology, Rollins College 8678, Winter Park, Florida, USA; 6 Adams School of Dentistry, University of North Carolina at Chapel Hill 2331, Chapel Hill, North Carolina, USA; 7 Department of Statistics, Santa Clara University 7162, Santa Clara, California, USA; 8 Department of Biology, Oregon State University 2694, Corvallis, Oregon, USA; 9 Department of Biology, Scripps College 31752, Claremont, California, USA; 10 Department of Molecular Microbiology and Immunology, OHSU School of Medicine 89020 , Portland, Oregon, USA; 11 Department of Oral and Molecular Microbiology, Osaka University Graduate School of Dentistry 595081, Suita, Osaka, Japan; 12 Department of Fundamental Microbiology, Faculty of Biology & Medicine, University of Lausanne 27213, Lausanne, Switzerland; 13 Department of Pediatrics, UC San Diego School of Medicine, La Jolla, California, USA; 14 Skaggs School of Pharmacy and Pharmaceutical Sciences, University of California San Diego 8784, La Jolla, California, USA; Indian Institute of Technology Bombay, Mumbai, Maharashtra, India

**Keywords:** *Streptococcus*, fatty acid biosynthesis

## Abstract

The genus *Streptococcus* contains some of the most important commensals and pathogens of the human microbiome. To obtain the fatty acids required for cell membranes, *Streptococcus* either produce fatty acids *de novo* through the fatty acid biosynthesis (*fab*) pathway or uptake host fatty acids through the fatty acid kinase (*fak*) pathway. Although both the *fab* and *fak* pathways represent potential therapeutic targets to prevent or treat infection*,* progress is limited because of an incomplete understanding of taxon-to-taxon variability in streptococcal lipid metabolism. Here, we examined the role of *de novo* monounsaturated fatty acid (MUFA) synthesis in physiology and virulence-associated traits in *Streptococcus mutans, Streptococcus pyogenes,* and *Streptococcus pneumoniae,* three major pathogens that cause disease at distinct body sites. In all three species, deletion of *fabM* abolished MUFA production and caused severe growth defects, decreased stress tolerance, increased antibiotic susceptibility, and defects in cell viability, morphology, and division. In *S. mutans,* loss of *fabM* also markedly reduced competence signaling and production of the mutacin IV bacteriocin. Deletion of *fabM* increased susceptibility to killing by human neutrophils in *S. mutans* and *S. pneumoniae,* but not *S. pyogenes*. Together, these findings illustrate that MUFA synthesis is broadly important for streptococcal physiology and cell membrane homeostasis, while its contribution to pathogenesis is strongly species- and context-dependent, providing leads to guide the development of novel therapeutic and/or preventative strategies.

**IMPORTANCE:**

*Streptococcus* spp. exert profound effects on human health, with several species causing significant morbidity and mortality. Although streptococcal fatty acid biosynthesis and utilization are attractive metabolic targets for development of therapeutics, this opportunity is vexed by an incomplete understanding of taxon-to-taxon variability in lipid metabolism. In this study, the role of *de novo* monounsaturated fatty acid (MUFA) synthesis in physiology and virulence-associated traits was examined in *Streptococcus mutans, Streptococcus pyogenes,* and *Streptococcus pneumoniae,* three major pathogens that cause disease at distinct body sites. MUFA synthesis was important for stress and antibiotic tolerance across all three species, while its impact on virulence was species- and context-dependent. Overall, these discoveries provide leads to guide the development of novel therapeutic and/or preventative strategies.

## INTRODUCTION

The genus *Streptococcus* includes some of the most important pathogens and commensals of the human microbiota, inhabiting diverse niches and exerting profound effects on host health and disease. *Streptococcus pyogenes* (group A *Streptococcus*), *Streptococcus agalactiae* (group B *Streptococcus*), and *Streptococcus pneumoniae* all appear on both the 2024 WHO Priority Pathogens List and the CDC Antibiotic Resistance Threats Report. To persist in their ecological niches and, in the case of pathogenic species, cause disease, streptococci must withstand a range of environmental stresses (1–4). As gram-positive bacteria, *Streptococcus* spp. are surrounded by a cell envelope consisting of a single cytoplasmic membrane (i.e., lipid bilayer) and a thick peptidoglycan cell wall; many species *additionally* produce a capsule that is critical for virulence (5).

The lipid bilayer of the cytoplasmic membrane is composed of diverse lipid species, typically consisting of one to four fatty acid chains attached to a head group (6). Fatty acid chains may vary in length and saturation. Saturated fatty acids (SFAs) adopt relatively straight configurations that allow tight packing and reduce membrane fluidity, whereas unsaturated fatty acids contain one or more double bonds that may introduce bends into the hydrocarbon chain, increasing steric hindrance and membrane fluidity (6). In *Streptococcus* spp.*,* fatty acids can either be synthesized *de novo* via the fatty acid biosynthesis (*fab*) operon (7) or incorporated from the environment through the fatty acid kinase (*fak*) system (8–10). *De novo* synthesis in streptococci is limited to SFAs and monounsaturated fatty acids (MUFAs). These fatty acids are attached to lipid head groups that, in *Streptococcus* spp., include glycerophosphates, sugars, and amino acids (11) through the *pls* machinery (12). By mixing and matching different fatty acid chains and head groups, bacteria can modulate membrane fluidity, curvature, and charge (6), thereby promoting survival under environmental stresses such as temperature extremes, acid stress, reactive oxygen species, and antibiotic exposure.

Previous research demonstrated that *Streptococcus gordonii, S. salivarius, S. mutans,* and *Lactobacillus casei* increase the proportion of MUFAs in their membranes in response to environmental acidification, largely at the expense of SFAs (13). In *S. mutans,* a similar shift toward increased MUFA content occurs under oxidative stress (14, 15). MUFAs are synthesized *de novo* in streptococci via the FabM enolyl-ACP isomerase, which was initially characterized biochemically using purified protein encoded by the *S. pneumoniae* gene sequence (16) and later through genetic disruption in *S. mutans* (17). In *S. mutans,* deletion of *fabM* resulted in membranes composed entirely of SFAs, accompanied by impaired tolerance to acid and oxidative stress and markedly reduced virulence in a rat model of dental caries (17, 18). In contrast, deletion of *fabM* (as well as ∆*fabF* and ∆*fabMF*) in *S. agalactiae* did not attenuate virulence in a neonatal mouse sepsis model (19). This discrepancy was hypothesized to reflect differences in the concentration and composition of environmental fatty acids available at distinct infection sites as fatty acid levels in saliva and dental plaque are substantially lower than those in blood (19).

These observations raised important questions regarding the suitability of fatty acid biosynthesis enzymes as therapeutic targets. The *fab* enzymes differ substantially from their eukaryotic counterparts, making them attractive candidates for selective inhibition (19–21). However, the ability of some streptococci to bypass *de novo* fatty acid synthesis by scavenging host-derived fatty acids through the *fak* system has complicated this paradigm. Subsequent studies have highlighted that the efficacy of Fab inhibitors (e.g., platensimycin) is highly dependent on both bacterial species and the infection context, including the availability of exogenous fatty acids at the infection site (21–24).

In this study, we further investigated the role of *de novo* MUFA biosynthesis in streptococcal physiology and virulence by examining *fabM* deletion mutants in *S. mutans, S. pyogenes,* and *S. pneumoniae,* three pathogens adapted to distinct anatomic niches. While *S. mutans* is a primary etiologic agent of dental caries and occasionally endocarditis (1), *S. pyogenes* causes diseases ranging from pharyngitis and impetigo to necrotizing fasciitis and toxic shock syndrome (4), and *S. pneumoniae* is a leading cause of pneumonia, otitis media, and meningitis (3). We show that, similar to *S. mutans*, deletion of *fabM* in *S. pyogenes* and *S. pneumoniae* abolishes MUFA production and severely impairs growth, phenotypes that could be rescued to varying degrees by exogenous unsaturated fatty acids. Across all three species, *fabM* deletion increases susceptibility to environmental stresses and antibiotics and is associated with defects in cell division, morphology, and viability.

## RESULTS

### Deletion of *fabM* in *S. mutans, S. pyogenes,* and *S. pneumoniae* eliminates production of UFAs and significantly impairs growth

**Fig 1:**
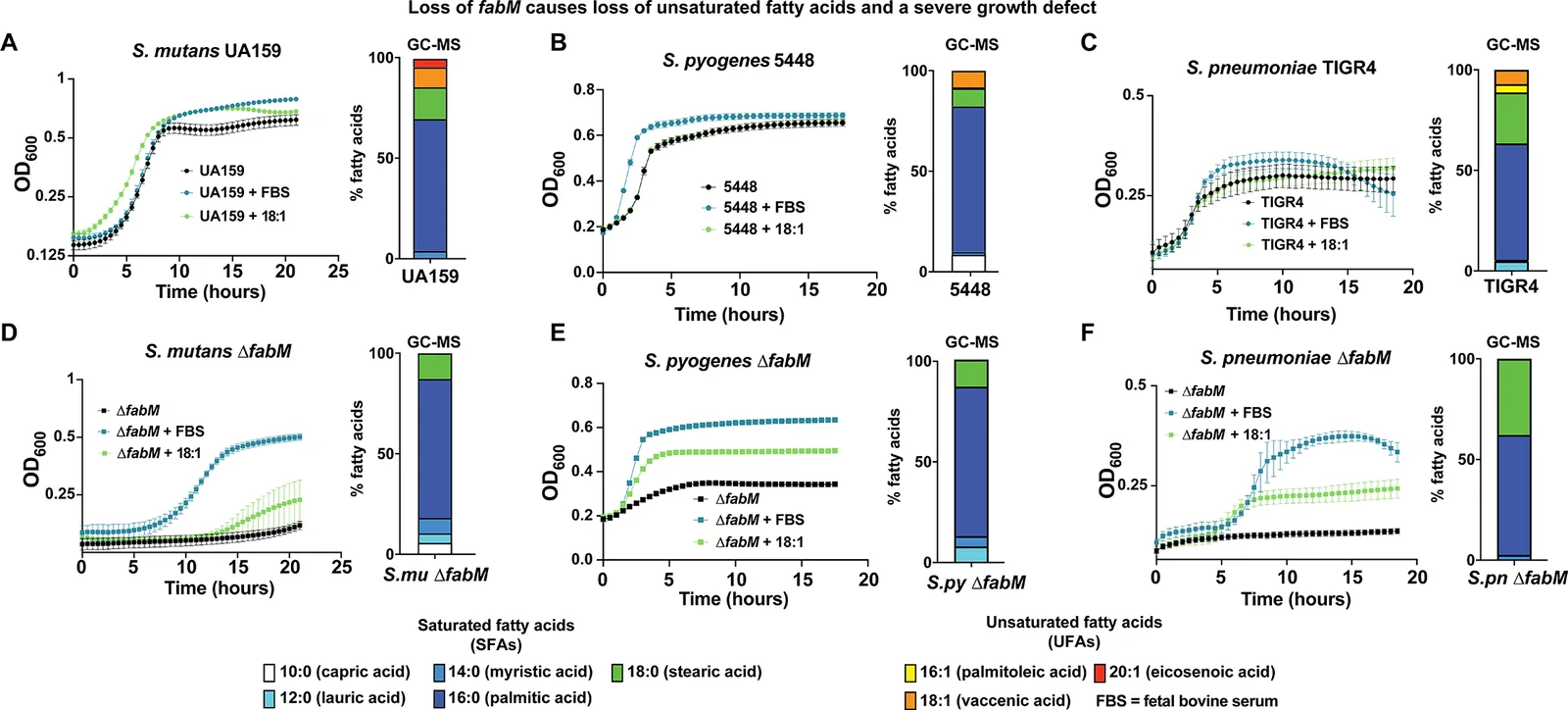
∆*fabM* strains have impaired growth and do not produce MUFAs. Growth curves of the indicated parental strains (**A–C**) or ∆*fabM* mutant strains (**D–F**) in Dulbecco’s modified Eagle medium (DMEM) with and without supplementation of either 10% FBS fetal bovine serum (FBS) or 10 µg/mL vaccenic acid (18:1cis11). Bar graph insets to the right of each growth curve display the relative abundances of fatty acids present in the cognate strain at the end of the growth curve in the unsupplemented DMEM, as determined by GC-MS. The legend for these bar charts is at the bottom of the figure.

A *fabM* deletion mutation in *S. mutans* (*Sm∆fabM*) was constructed as part of the single-gene knockout library in *S. mutans* UA159 described in (14). This mutant was significantly impaired in overall growth, defective in biofilm formation, and markedly less tolerant to acid and oxidative stress (14). Consistent with prior reports using an insertional *fabM*::erm mutant (17, 25), deletion of *fabM* abolished the production of UFAs in *S. mutans* (Fig. 1). Complement strains of *S. mutans fabM* have been described previously and restored MUFA production, growth, and acid and oxidative stress tolerance to a large degree (17, 26). The *fabM* complement strain described as UR300 in reference 26 was used here and designated *Smu-fabM-C*.

To assess whether *de novo* MUFA biosynthesis plays a similar role in streptococcal pathogens adapted to cause infection at other body sites—and therefore exposed to distinct types and concentrations of environmental fatty acids—single-gene *fabM* deletion mutants were also created in the highly virulent M1T1 serotype *S. pyogenes* strain 5448 (*Spy∆fabM*) and capsule serotype 4 *S. pneumoniae* strain TIGR4 (*Spn∆fabM*). As observed for *Smu∆fabM,* both *Spy∆fabM* and *Spn∆fabM* failed to produce UFAs and exhibited significant growth defects in unsupplemented media (Fig. 1). As described in the *Materials and Methods*, there were single-nucleotide polymorphisms (SNPs) identified in the *Spy∆fabM* (*gdpP*) and *Spn∆fabM (rrgA*) strains that may have impacted the phenotypes. Therefore, complemented strains of *Spy∆fabM* (*Spy-fabM-C*) and *Spn*∆*fabM* (*Spn-fabM-*C) were also constructed. While *Spn-fabM-C* fully restored MUFA production and suppressed the impaired growth phenotype, *Spy-fabM-C* only partially restored the *Spy∆fabM* MUFA production and growth phenotypes (Fig. S1). It is possible that only partial complementation in *Spy-fabM-C* could either be because of an additional SNP acquired in the *fabT* transcriptional regulator and/or polar effects of the kanamycin resistance marker upstream of *fabM* in the complement strain, possibly leading to reduced expression. In any case, compared to the parent ∆*fabM* strains, both complement strains had improved growth and produced MUFAs, strongly indicating that FabM was responsible for these phenotypes. It is also possible that deletion of ∆*fabM* in *S. pyogenes* and *S. pneumoniae* leads to UFA autotrophy and the detected SNPs in *Spy∆fabM* and *Spn∆fabM* allow for some growth. Further investigation of the impact of these SNPs on physiology and virulence phenotypes is in progress.

**Fig 2:**
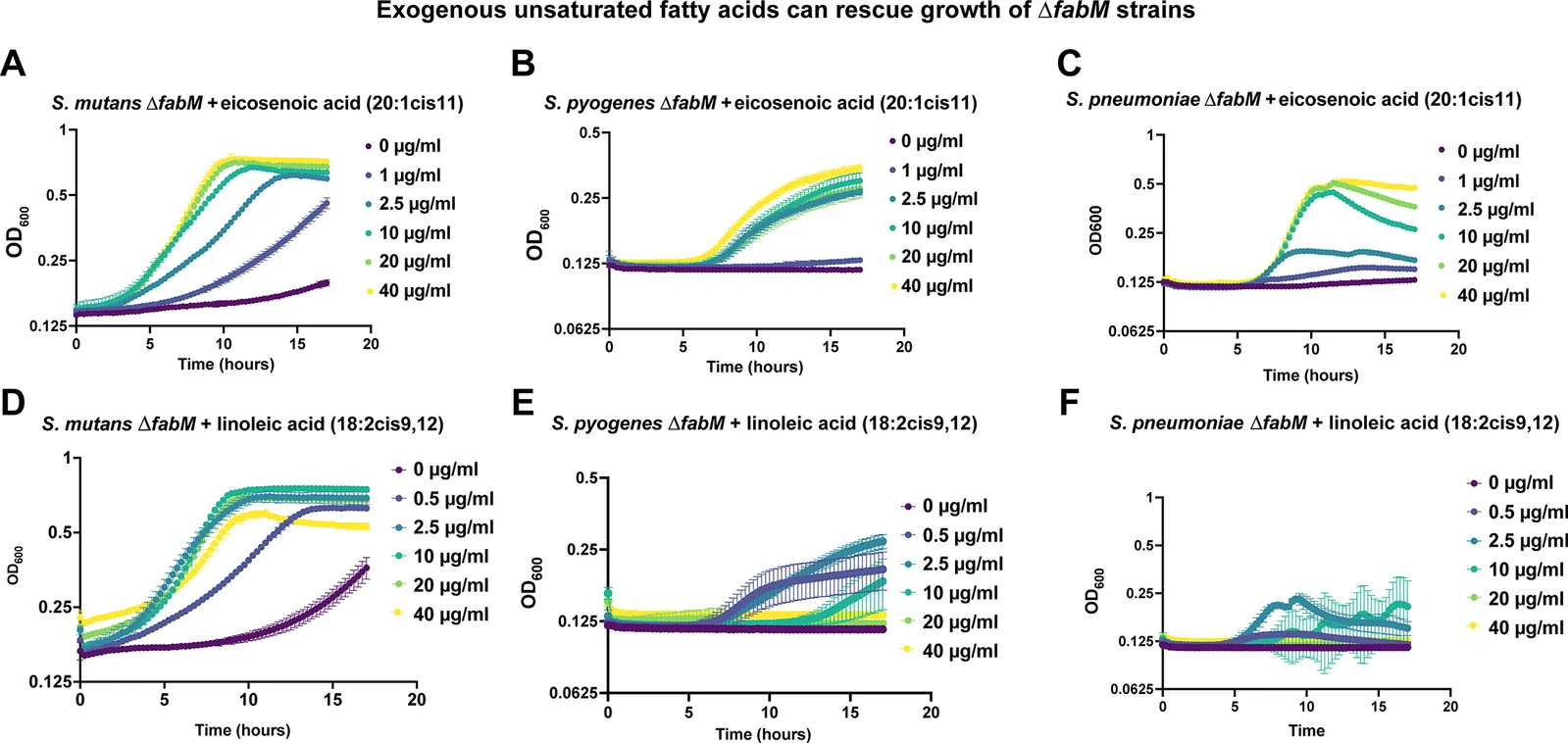
Exogenous monounsaturated and polyunsaturated fatty acids can rescue growth of ∆*fabM* strains. Growth curves of the indicated strains with eicosenoic acid (**A–C**) or linoleic acid (**D–F**) supplements at the indicated concentrations.

In all three species, the growth defect of the ∆*fabM* strains could be rescued nearly to parental levels by supplementation of the growth media with fetal bovine serum (FBS), which contains both MUFAs and PUFAs. Growth was also improved by addition of purified vaccenic acid (18:1cis11), typically the most abundant UFA synthesized *de novo* by streptococci (13, 17, 27) (Fig. 1). The ability of other unsaturated fatty acids to rescue growth of the ∆*fabM* strains was also examined (Fig. 2; Fig. S2). In all three species, the MUFAs palmitoleic acid (16:1cis9), oleic acid (18:1cis9), and eicosenoic acid (20:1cis11) rescued growth in a dose-dependent manner, with maximal rescue observed at concentrations up to 40 µg/mL for *Smu*∆*fabM* and *Spn*∆*fabM* and up to 10 µg/mL for *Spy*∆*fabM* (Fig. 2; Fig. S2). The diene PUFA linoleic acid (18:2cis 9,12), which is abundant in human blood, also rescued growth but over a narrower concentration range. Linoleic acid rescued growth of *Smu*∆*fabM* in a dose-dependent manner up to 20 µg/mL, whereas higher concentrations were inhibitory. In contrast, growth of *Spy*∆*fabM* and *Spn*∆*fabM* was rescued only at low concentrations ( <2.5 µg/mL) with higher concentrations strongly inhibitory (Fig. 2; Fig. S2).

Streptococci are not known to synthesize PUFAs *de novo* but can incorporate exogenous PUFAs through the *fak* system. Notably, linoleic acid rescued growth of *Smu*∆*fabM,* despite the absence of *fakB3,* the gene encoding the PUFA-incorporating FakB component characterized in *S. pneumoniae* (8). This finding suggests that *S. mutans may* encode an alternative mechanism for PUFA incorporation. Although *S. pyogenes* encodes a *fakB3* homolog (28)*,* the role of this gene in PUFA utilization for this species has not been directly examined. As expected, provision of the SFA stearic acid (18:0) did not rescue growth of any ∆*fabM* strain (data not shown).

### Deletion of *fabM* in *S. mutans, S. pyogenes,* and *S. pneumoniae* increases susceptibility to acid stress, oxidative stress, and antibiotics

An insertional mutation of *fabM* in *S. mutans* was previously shown to impair tolerance to environmental stresses such as acid and oxidative stress (17). To determine whether deletion of *fabM* produces similar phenotypes across streptococcal species, the stress tolerance of *∆fabM* strains and their cognate parent strains was examined under conditions of temperature stress, acid stress, oxidative stress, and antibiotic exposure.

**Fig 3:**
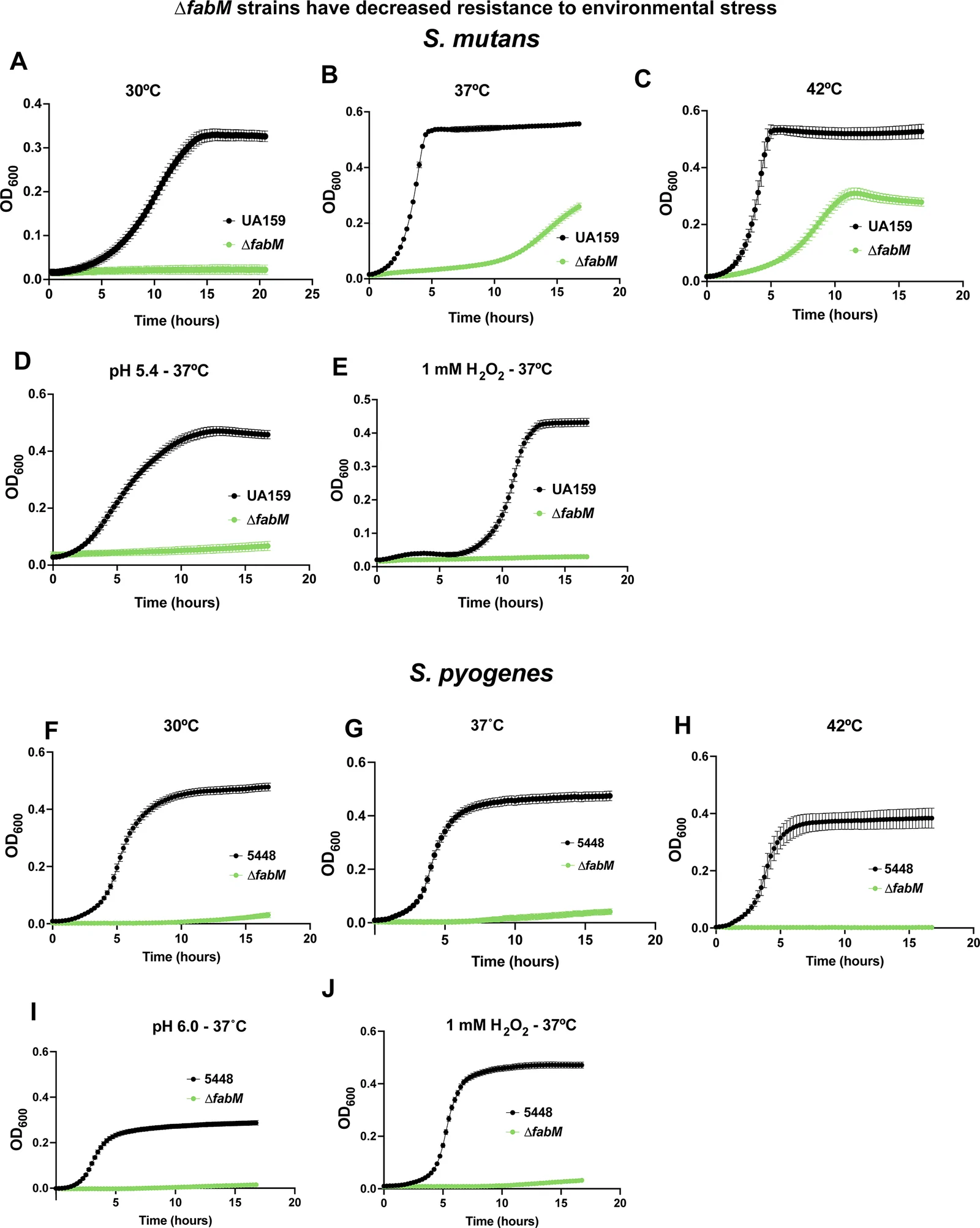
∆*fabM* strains are sensitive to adverse temperature, acid, and oxidative stress. Growth curves of the indicated strains of *S. mutans* (**A–E**) or *S. pyogenes* (**F–J**) grown in Todd-Hewitt broth (THB) at the indicated temperatures (**A–H**), grown at 37°C in THB buffered to the indicated acidic pH (**D–I**), or grown at 37°C in THB + 1 mM H_2_O_2_ (**E–J**). *S. pneumoniae* phenotypes under these conditions were the same as *S. pyogenes and* are shown in Fig. S3.

In *S. mutans, Smu*∆*fabM* exhibited no detectable growth at 30°C, while growth at 42°C was improved relative to growth at 37°C (Fig. 3; Fig. S3). Growth of *Smu*∆*fabM* was also reduced to nearly undetectable levels at pH 5.4 and in the presence of 1 mM H_2_O_2_, consistent with previous reports describing the *Smu*∆*fabM* phenotype. In contrast, *Spy∆fabM* and *Spn∆fabM* exhibited only slightly reduced growth at 30°C relative to 37°C and showed little to no growth at 42°C (Fig. 3; Fig. S3). All three ∆*fabM* strains exhibited pronounced sensitivity to acid stress, with little to no growth at low pH (Fig. 3; Fig. S3).

*Spy∆fabM* and *Spn∆fabM* were less sensitive to H_2_O_2_ than *Smu∆fabM*. This difference is likely attributable to the fact that *S. pyogenes* and *S. pneumoniae* can produce much higher levels of H_2_O_2_ than *S. mutans* and therefore encode several mechanisms to tolerate oxidative stress (1, 29–31). To assess whether loss of MUFA synthesis also alters antibiotic susceptibility, minimum inhibitory concentrations (MICs) were determined for antibiotics representing multiple mechanistic classes: ampicillin, daptomycin, polymyxin B, and spectinomycin. In all three species, MICs for daptomycin, polymyxin B, and spectinomycin were reduced in the ∆*fabM* strains compared to their cognate parent strains (Table 1). For ampicillin, MICs were reduced in the ∆*fabM* strains of *S. mutans* and *S. pyogenes*, whereas for *S. pneumoniae,* both TIGR4 and *Spn∆fabM* exhibited MICs below the limit of detection (Table 1).

**TABLE 1:** MICs of ampicillin, daptomycin, polymyxin B, and spectinomycin for the indicated strains.

Antibiotic	MIC for WT	MIC for ∆fabM
Ampicillin		
*S. mutans*	0.06	0.16
*S. pyogenes*	0.004	<0.002
*S. pneumoniae*	<0.002	<0.002
Daptomycin		
*S. mutans*	50	25
*S. pyogenes*	5	<0.313
*S. pneumoniae*	1	<0.25
Polymyxin B		
*S. mutans*	32	4
*S. pyogenes*	16	6
*S. pneumoniae*	48	32
Spectinomycin		
*S. mutans*	25	<0.78
*S. pyogenes*	6.25	<0.78
*S. pneumoniae*	3.125	<0.78

Taken together, these data indicate that loss of *fabM* and the absence of *de novo* MUFA production broadly sensitize streptococcal pathogens to environmental stresses and multiple classes of antibiotics, consistent with compromised membrane and cell envelope homeostasis.

### Mutacin production and competitive fitness versus neighboring commensals is impaired in *Smu*∆*fabM*

**Fig 4:**
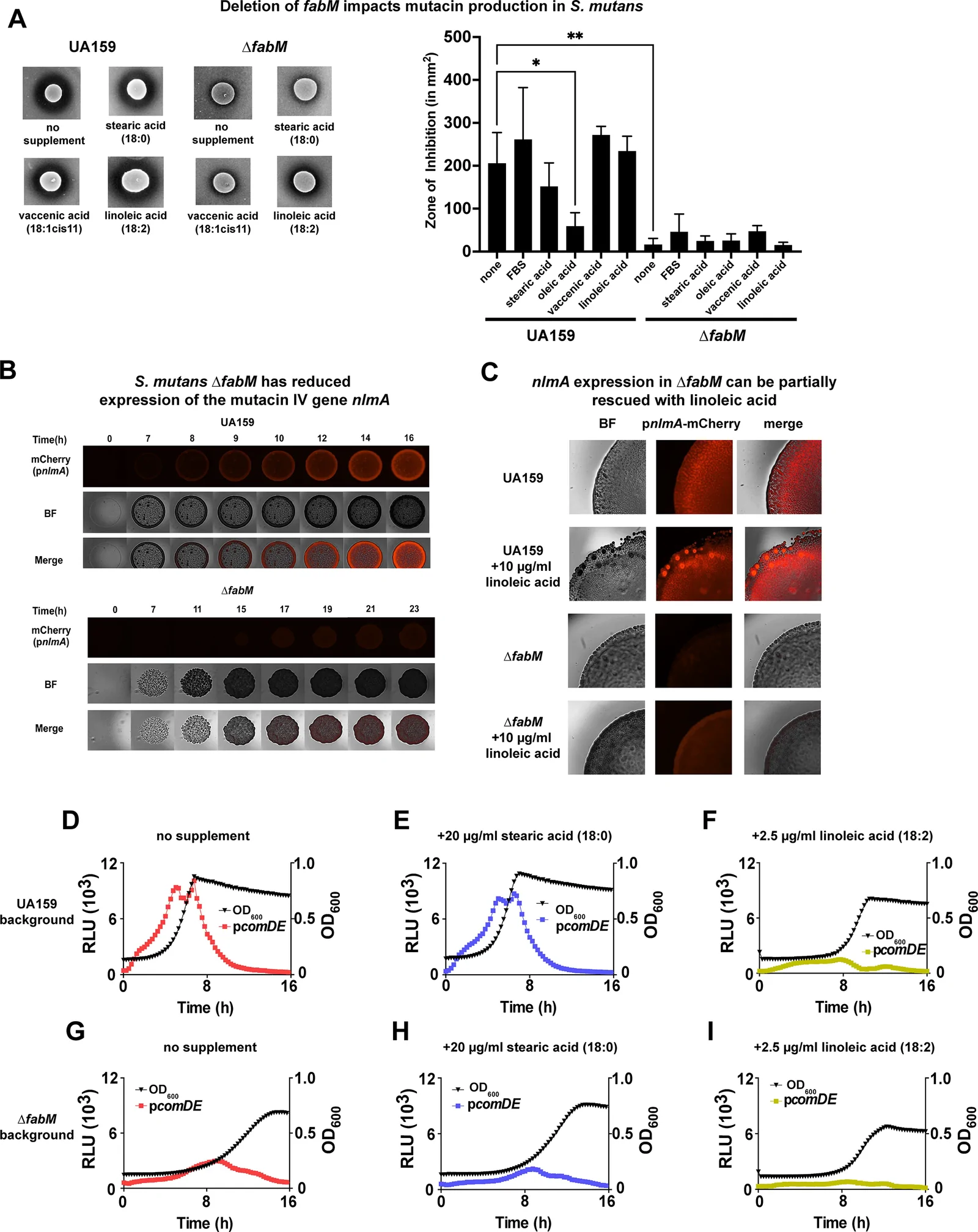
Deletion of *fabM* in *S. mutans* impacts mutacin production and competence signaling. Panel **A** includes representative photos of the zone of inhibition produced by 8 µL colonies of the indicated strain spotted on THB agar with the indicated supplements with a *S. sanguinis* overlay in soft agar. The bar graph on the right side of Panel **A** is a quantification of three experimental replicates of the indicated strain/conditions of the same competition assay between *S. mutans* and *S. sanguinis* displayed in the Panel **A** photos. Panel **B** shows representative photos of a time course of growth of a p*nlmA-*mCherry reporter in either a UA159 or *Smu*∆*fabM,* as indicated. BF, bright field. Panel **C** shows representative images of the p*nlmA-*mCherry reporter in the indicated background strain, with or without supplementation of 10 µg/mL linoleic acid, as indicated. Panels **D–I** are superimposed growth curves and luminescence produced by a p*comDE-*luciferase reporter, with the indicated supplements, in either a UA159 (**D–F**) or ∆*fabM* (**G–I**) background.

Many *Streptococcus* species produce bacteriocins that inhibit the growth of neighboring bacteria, often other streptococci inhabiting the same ecological niche. To assess whether deletion of *fabM* impacts competitive fitness, *Smu*∆*fabM* was examined in a standard competition assay on THB agar commonly used to measure inter-streptococcal antagonism. *Smu*∆*fabM* was significantly less effective than the parent UA159 strain at inhibiting growth of the oral commensals *Streptococcus gordonii, S. sanguinis,* and *S. mitis* (shown in Fig. 4A for *S. sanguinis*; data not shown for *S. gordonii* and *S. mitis*). Although supplementation of the growth medium with MUFAs and/or PUFAs partially improved competitive inhibition by *Smu*∆*fabM*, these effects did not reach statistical significance due to assay variability (Fig. 4A). Interestingly, a similar increase in inhibitory activity was observed for the UA159 parent strain upon fatty acid supplementation (Fig. 4A). Complementation of ∆*fabM* in *S. mutans* reversed the mutacin-deficient phenotype of the *Smu*∆*fabM* strain, as expected (Fig. S4).

Because *S. mutans* mutacin IV is the primary bacteriocin responsible for inhibition of the tested commensal *Streptococcus* spp. in UA159, we examined the expression of the *nlmA* component of mutacin IV using a P*_nlmA_-mCherry* transcriptional reporter. In the UA159 background, robust mCherry fluorescence was readily observed overlaid with colonies grown on agar plates (Fig. 4B). In contrast, *Smu*∆*fabM* exhibited negligible mCherry fluorescence, indicating a marked reduction in *nlmA* expression. Expression of mCherry in the ∆*fabM* background could be rescued to a modest degree by provision of linoleic acid (Fig. 4B and C).

In *S. mutans,* mutacin production is typically controlled by the two-component quorum sensing system ComDE, which senses the competence-stimulating peptide CSP (1). To determine whether deletion of *fabM* affects ComDE signaling, P*_comDE_-*luciferase reporter strains were generated in both the UA159 and *Smu*∆*fabM* backgrounds. The expression of *comDE* was significantly reduced in *Smu*∆*fabM* grown in unsupplemented media as well as media supplemented with stearic acid (Fig. 4D, G, E and H), consistent with the observed reduction in *nlmA* expression and mutacin production. Unexpectedly, addition of linoleic acid reduced, rather than increased, *comDE* expression in both the UA159 and ∆*fabM* backgrounds (Fig. 4F and I). This contrasted with the partial restoration of *nlmA* expression in the ∆*fabM* background upon linoleic acid supplementation, suggesting that linoleic acid may decouple *nlmA* expression from canonical ComDE-dependent regulation.

The *S. pyogenes* and *S. pneumoniae* strains examined in this study did not inhibit growth of the tested commensal streptococci under the assay conditions used (data not shown). Although *S. pneumoniae* produces Blp bacteriocins similar to the *S. mutans* mutacins, those produced by the TIGR4 are not functional and also lack a competence-associated regulatory region (32, 33).

Because *S. pneumoniae* and *S. pyogenes* typically cause α and β hemolysis, respectively, the impact of *fabM* deletion on hemolysis was also examined using THB agar supplemented with blood. In both cases, hemolysis was not affected, potentially reflecting complementation of the *∆fabM* phenotype by UFAs present in blood (data not shown).

### ∆*fabM* strains exhibit disrupted cell morphology and altered CFU/OD_600_ ratios

**Fig 5:**
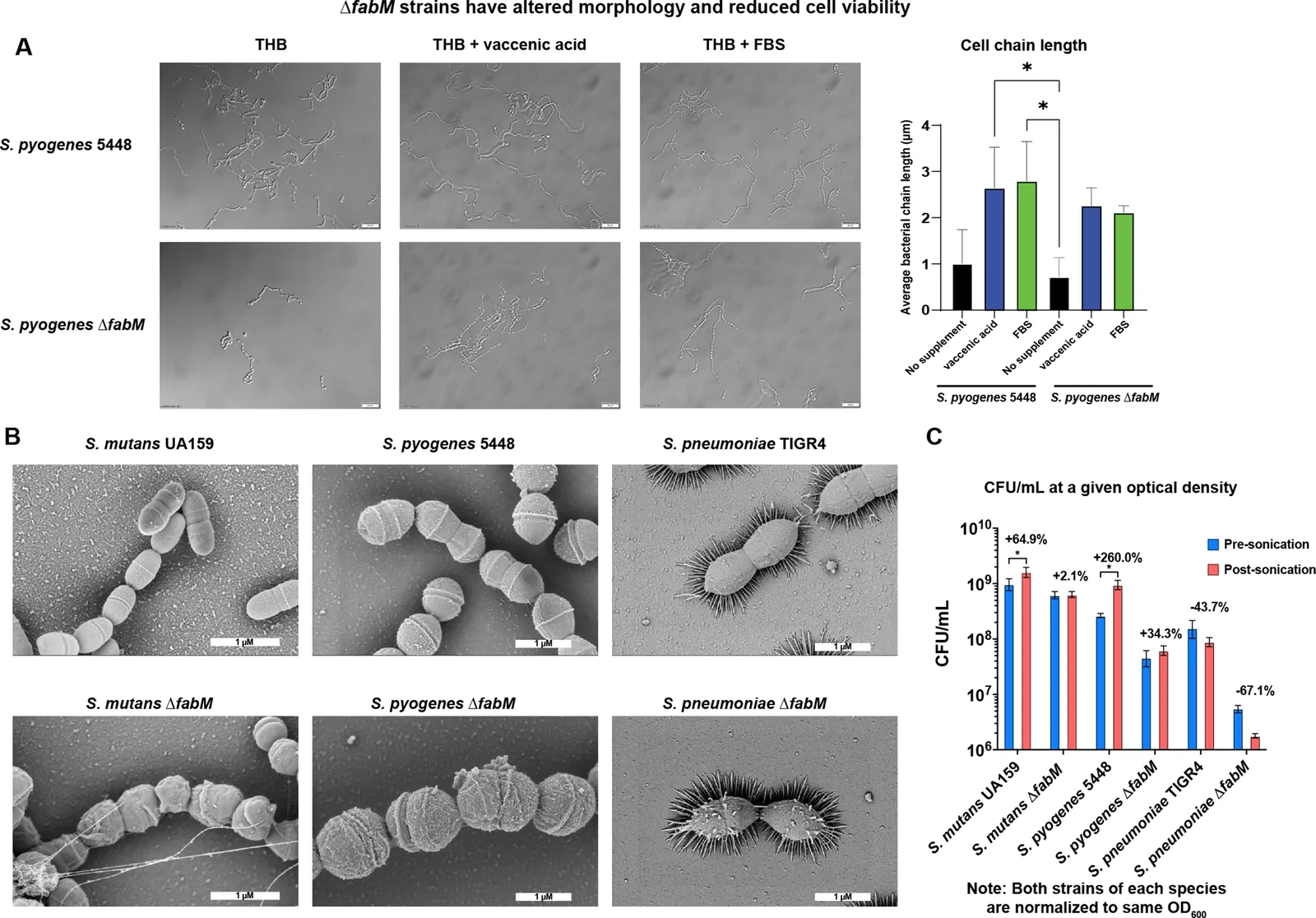
Deletion of *fabM* impacts cell morphology. Panel **A** shows representative images from light microscopy at 63× magnification of the indicated strain and growth condition; the inset to the right is a bar graph illustrating the quantitation of the effect of *fabM* deletion and/or UFA supplementation on cell chain length in *S. pyogenes*. *denotes statistical significance (*P* < 0.05) between the indicated groups of samples based on Tukey’s Honestly Significant Differences test following a one-way analysis of variance (ANOVA). Panel **B** shows representative images of scanning electron microscopy of the indicated strains. Note the asymmetry of cells and divisomes in the ∆*fabM* strains, as well as additional membrane blebs in the *Spn∆fabM* strain, specifically. “Spikes” surrounding the *S. pneumoniae* cells are believed to be an artifact of the capsule and fixation process. Images were taken at 80,000×. Panel **C** is a bar graph of the colony-forming unit (CFU)/mL of the indicated strain, either pre- or post-sonication, as indicated. Both strains of each species are normalized to the same OD_600_. *denotes statistical significance (*P* < 0.05) between the indicated groups of samples based on Tukey’s Honestly Significant Differences test following a one-way ANOVA.

Initial examination of the ∆*fabM* strains by light microscopy revealed abnormal cell morphology in all three species. Although chains of cells formed by the *S. mutans* and *S. pyogenes* ∆*fabM* strains appeared shorter than those of their cognate parent strains, this difference was not statistically significant due to substantial variability in chain length (Fig. 5A; Fig. S2). Supplementation of the growth medium with UFAs (vaccenic acid or FBS) resulted in significantly longer chains in *S. pyogenes* 5448, but not in the *Spy∆fabM* strain (Fig. 5A). Deletion of *fabM* did not measurably impact chain length in *S. pneumoniae,* which typically forms very short chains under the conditions tested (Fig. S2).

While there were no significant qualitative differences in cell lengths or widths, the ∆*fabM* strains of all three species displayed pronounced morphological abnormalities compared to their respective parent strains when viewed at higher magnification using scanning electron microscopy (Fig. 5B). In *Smu*∆*fabM* and *Spy*∆*fabM*, cells frequently exhibited asymmetrical septa and, in some cases, appeared to initiate division without completing septation, suggestive of impaired cell division (Fig. 5B). In *Spn*∆*fabM,* cells appeared more ovoid and exhibited more membrane blebs relative to TIGR4 (Fig. 5B). Both *S. pneumoniae* strains exhibited spike-like structures surrounding the cells, which are believed to represent fixation artifacts associated with the capsule (34).

During routine normalization of cultures based on optical density (OD_600_), an inconsistency became apparent between OD_600_ and viable cell counts in the ∆*fabM* strains. Quantification revealed a significant reduction in the ratio CFU/OD_600_ ratio for all three ∆*fabM* strains compared to their cognate parent strains: a 1.67-fold decrease for *Smu*∆*fabM*, a 3.86-fold decrease for *Spy*∆*fabM,* and a 23.63-fold decrease for *Spn*∆*fabM* (Fig. 5C). These findings indicate that, for a given culture density, a substantially smaller proportion of cells in the Δ*fabM* cultures are capable of forming colonies. Therefore, either the cells are dying or daughter cells are being created which are not viable for further division (which might be suggested by the aberrant cell septa observed in the SEM).

Because *Streptococcus* spp. often form chains, sonication is routinely used prior to CFU enumeration to break up chains and improve quantification. After sonication, CFU counts increased dramatically for the UA159 and 5448 parent strains, whereas only modest increases were observed for their corresponding ∆*fabM* strains (Fig. 5C). This difference is consistent with the shorter average chain length observed in the ∆*fabM* strains and/or increased sensitivity of these strains to sonication. In contrast, sonication reduced CFU counts for both TIGR4 and *Spn*∆*fabM*, indicating that the sonication procedure employed was detrimental to *S. pneumoniae* viability, with the *Spn*∆*fabM* strain exhibiting greater sensitivity (Fig. 5C).

Taken together, these results indicate that *de novo* MUFA synthesis is required for proper cell morphology and division in streptococci. Loss of *fabM* results in defective septation, abnormal cell shape, and a marked reduction in the fraction of viable cells within the population, consistent with compromised membrane function and cell envelope integrity.

### *Smu*∆*fabM* and *Spn*∆*fabM,* but not *Spy∆fabM,* are more susceptible to neutrophil killing

**Fig 6:**
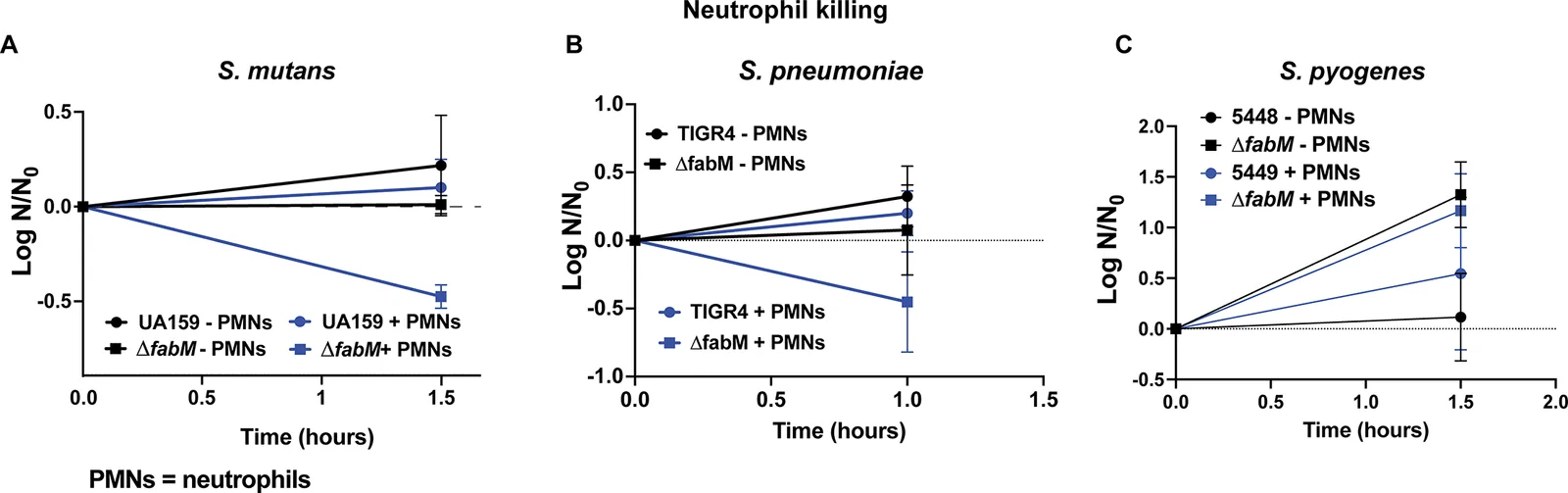
Deletion of *fabM* impacts susceptibility to neutrophil killing for *S. mutans* and *S. pneumoniae*. (**A–C**) Graphs indicating survival of the indicated species and strain in either the absence or presence of neutrophils, as indicated.

Because loss of MUFA synthesis is expected to change the properties of the cell envelope, which may in turn influence susceptibility to host immune defenses, the ∆*fabM* strains and their cognate parent strains were examined in a neutrophil killing assay using primary human neutrophils. *Smu*∆*fabM* and *Spn*∆*fabM* were readily killed by neutrophils, whereas the corresponding parent strains were not susceptible and exhibited CFU counts comparable to control conditions lacking neutrophils (Fig. 6A). In contrast, neither *S. pyogenes* 5448 nor *Spy*∆*fabM* exhibited detectable susceptibility to neutrophil-mediated killing under the conditions tested (Fig. 6C).

## DISCUSSION

As the physical barrier separating the inside and outside of the cell, the membrane plays a key role in physiology, and its properties have wide-reaching impacts on how cells interact with their surroundings (6). *Streptococcus* spp. are able to either generate SFA and MUFA chains *de novo* through the *fab* operon (7) or use the *fak* system to incorporate exogenous fatty acids from the environment (8). The enoyl-ACP isomerase encoded by *fabM* is responsible for *de novo* biosynthesis of MUFAs, while *Streptococcus* are not known to produce PUFAs (16). Several species of *Streptococcus* are known to increase the proportion of MUFAs in their membrane in response to environmental acidification. However, it remains unknown how this adjustment is controlled or precisely why this response is protective (13, 35). In *S. mutans,* a *fabM* insertional mutant had no MUFAs in its membrane and exhibited impaired growth and acid tolerance, leading to reduced virulence in a rat model of dental caries (17, 18). This was in stark contrast to a study that showed that an *S. agalactiae* ∆*fabM* strain was fully virulent in a mouse neonatal sepsis model (19). The same study also showed full virulence of ∆*fabF* and ∆*fabMF* strains, which would presumably be deficient in all *de novo* fatty acid syntheses, relying entirely on incorporation of exogenous fatty acids for growth (19). The *fab* genes were initially touted as promising antibiotic targets due to their major structural differences from their eukaryotic counterparts (i.e., therefore inhibitors would likely have a low toxicity to mammalian cells) (36). However, the ability of certain species to circumvent the need for the *fab* genes by using exogenous fatty acids, as shown in the *S. agalactiae* study, brought the suitability of the *fab* genes as drug targets into question (37). Subsequent research has illustrated that the utility of *fab* inhibitors is highly dependent on both the bacteria in question and the environmental context (21, 38, 39).

The goal of this study was to further characterize the role of MUFA production in *Streptococcus* physiology and virulence by continuing to determine the role of *fabM* in *S. mutans* as well as explore the impact of ∆*fabM* deletion in *S. pyogenes* and *S. pneumoniae. S. mutans, S. pyogenes,* and *S. pneumoniae* have evolved to cause disease in different environments, each of which contains different types and concentrations of fatty acids. Similar to what was observed previously in *S. mutans* (17), the ∆*fabM* strains in *S. pyogenes* and *S. pneumoniae* had severely impaired growth nearly to the point of auxotrophy, which could be rescued to varying degrees by exogenous UFA supplementation. A previous study also examined *fabM* null strains in *S. mutans* and *S. pneumoniae* and reported that the strains were auxotrophs, with no growth in unsupplemented media (40). Differences in observed phenotypes between this study and that study are likely due to the variability in isolates used and/or trace amounts of UFAs in the different growth media used. It is also possible that the SNPs identified in our *Spy∆fabM* and *Spn∆fabM* strains suppressed complete UFA auxotrophy in these isolates.

The observed lack of growth *of Smu∆*fabM at 30°C and improved growth at 42°C is plausible as a membrane composed entirely of SFAs would be more rigid (i.e., a higher melting point and less able to withstand colder temperatures). Indeed, other bacteria are known to increase the proportion of UFAs in their membranes in response to cold (41). With exclusively SFAs in *Sm∆fabM,* it also makes sense that growing at higher-than-normal temperatures would be less of an issue. In contrast, *Spy∆fabM* and *Spn∆fabM* exhibited some growth at 30°C and no growth at 42°C. This could possibly be due to these strains evolving to reside in different environments with slightly different temperatures, such as the respiratory tract and skin. Notably, *S. mutans* in general had more MUFAs than both *S. pyogenes* and *S. pneumoniae. S. mutans* also produced 20-carbon fatty acids, mainly eicosenoic acid (20:1), along with trace amounts of eicosanoic acid (20:0). These 20-carbon fatty acids were not observed in *S. pyogenes* or *S. pneumoniae*. Similar to *S. mutans,* the *Spy∆fabM* and *Spn∆fabM* strains were impaired in their ability to withstand acid stress, exhibiting no growth at pH 6, although *S. mutans* was more acid-tolerant overall, in line with previous observations (1). Oxidative stress in the form of H_2_O_2_ significantly impaired growth of *Smu∆fabM,* but not *Spn∆fabM* or *Spy∆fabM*. While *S. mutans* does not produce high concentrations of H_2_O_2_, *S. pneumoniae* and some strains of *S. pyogenes* produce copious H_2_O_2_ through enzymes such as lactate oxidase, the peroxide-forming NADH oxidase, and pyruvate oxidase and therefore have evolved to tolerate these higher concentrations (29–31). *S. pyogenes* and *S. pneumoniae* may use H_2_O_2_ to inhibit the growth of competitors, and H_2_O_2_ in *S. pneumoniae* is known to be involved in heme degradation (42, 43), capsule formation (44), pneumolysin production (45), MUFA production (35), and FabF oxidation (46). This also aligns with *S. mutans* containing significantly more MUFAs when grown in conditions of oxidative stress (15, 47). Across all three species, ∆*fabM* strains exhibited a general increase in susceptibility to antibiotics. Since a previous study identified that there were over 300 differentially abundant transcripts identified between *S. mutans* UA159 and *Sm∆fabM* (25)*,* there are a host of metabolic pathways likely to be affected and responsible for these phenotypes.

*Smu∆fabM* was significantly impaired in its ability to inhibit the growth of the commensal *Streptococcus*. As mutacin production in *S. mutans* is frequently controlled by the two-component ComDE quorum sensing system (1, 48), the impact of ∆*fabM* on *comDE* expression was examined. *comDE* expression was significantly reduced in the ∆*fabM* strain, which may explain the reduction in *nlmA* expression and mutacin IV production. However, addition of linoleic acid also reduced *comDE* expression, indicating that the rescue of *nlmA* expression observed upon addition of linoleic acid must have been independent of *comDE* signaling. The observation that linoleic acid inhibited the *comDE* system was especially interesting as this system is highly involved in virulence traits such as biofilm formation, competence, and toxin production (48). Although unsaturated fatty acids have been shown to impact quorum sensing in *Pseudomonas aeruginosa* (49) and *Acinetobacter baumannii* (50)*,* there have been no such reports in *Streptococcus*. However, it is notable that short-chain fatty acids impact *comDE* in *S. gordonii* (51) and that the BriC quorum sensing peptide regulates fatty acid biosynthesis in *S. pneumoniae* (52). The impact of long-chain SFAs and UFAs on *Streptococcus* quorum sensing warrants further study, which is currently in progress.

SEM indicated that while the cells of the parent strains had regular shapes with symmetrical septa and divisomes, most ∆*fabM* cells were asymmetrical—some with multiple septa, especially in *Smu∆fabM* and *Spy∆fabM*. In *Spn∆fabM,* cells appeared to have a shorter, more ovoid shape and had a greater number of membrane blebs compared to TIGR4. Collectively, these images indicate that the ∆*fabM* strains have a defect in cell division and/or morphology. In *S. pneumoniae,* it was reported that SFAs tended to colocalize with the cell-division machinery, while UFAs made up the other regions of the cell (53). It seems likely that in the absence of UFAs, membrane fluidity is abnormal, and the cell division machinery has difficulty localizing or operating properly. This seemed to translate to an abundance of cells in the cultures that were apparently not viable. Furthermore, when cultures of the cognate parent strains and ∆*fabM* strains were normalized to the same culture density, the number of CFU/mL of the ∆*fabM* strains was significantly lower, indicating that many of the cells in the ∆*fabM* strains are not viable. Sonication of the cells to break up the chains is commonly used prior to quantification of *Streptococcus* spp. by CFU plating. This sonication increased the number of CFUs in the parent strains of *S. mutans* and *S. pyogenes* but did so to a lesser extent in the ∆*fabM* strains, possibly due to the shorter chain length in the ∆*fabM* strains. Alternatively, the ∆*fabM* cells could be more sensitive to sonication, and the process may kill some of the cells. Notably, *S. pneumoniae* decreased in CFU/mL following sonication, indicating it may be more sensitive to the sonication protocol.

The *S. mutans* and *S. pneumoniae ∆fabM* strains were more susceptible to killing by human neutrophils, likely reflective of the general susceptibility to stress observed in the ∆*fabM* mutants. It is unclear exactly why the *S. pyogenes ∆fabM* strain was still resistant to neutrophil killing; however, *S. pyogenes* is known to employ several mechanisms to avoid this fate, including evasion of phagocytosis, resistance to antimicrobial peptides, and escape from neutrophil extracellular traps (29, 54).

Research further examining the ability of *S. mutans* to utilize PUFAs while lacking a *fakB3* gene, as well as further study of the impact of exogenous fatty acids on *Streptococcus* competence signaling and virulence, are currently in progress. Overall, this study also suggested that UFAs play an important role in the organization and function of the cell division machinery. As data accumulate showing that the effectiveness of fatty acid biosynthesis inhibitors is highly dependent on species and disease context, examining further relevant situations such as lung infection by *S. pneumoniae* or skin infection by *S. pyogenes* is likely to open the door to novel, context- and pathogen-specific preventative and therapeutic strategies.

## MATERIALS AND METHODS

### Bacterial strains and growth conditions

All strains, plasmids, and primers used in this study are listed in Table S1. All *Streptococcus* strains were maintained on Todd-Hewitt broth (THB) agar plates (BD/Difco, Franklin Lakes, NJ) at 37°C in a 5% (vol/vol) CO_2_–95% air environment. The *S. mutans* strains UA159 (55), *Smu*∆*fabM* (previously designated MU1592) (14, 17)*,* and *Smu-fabM-C* (previously designated UR300) (26) have been described previously. *Smu-fabM-C* faithfully restored production of MUFAs, and most other phenotypes of the *fabM* deletion (17) (26).

### Generation of recombinant strains: *S. pyogenes* ∆*fabM*

Genomic DNA from *S. pyogenes* 5448 (56) was isolated using the Quick-DNA Fungal/Bacterial Miniprep Kit (Zymo, Inc.) according to the manufacturer’s instructions; 500 bp upstream of *fabM* was amplified using the PCR primer pair Spy-fabM-LF-F and Spy-fabM-LF-R, 500 bp downstream of *fabM w*as amplified using PCR primer pair Spy-fabM-RF-F and Spy-fabM-RF-R, and the erythromycin resistance gene was amplified using the PCR primer pair hsdR-ery-F and hsdR-ery-R using chromosomal DNA of strain NV6 as a template (57). PCR products were purified using the DNA Clean and Concentrator Kit (Zymo, Inc.). The three fragments were then ligated together using Golden Gate Assembly with T4 DNA ligase and BsaI-HF2 (New England Biolabs). The ligation was performed in a thermocycler with the following protocol: 1.5 min at 37°C; 3 min at 16°C; repeated 50×; 5 min at 37°C; 10 min at 80°C; hold at 4°C. The ligation product was gel-purified and further amplified using the PCR primer pair Spy-fabM-LF-F and Spy-fabM-RF-R. One microgram of the purified PCR product was added to 50 µL NV2 competent cells (*S. pyogenes* 5448 containing the pAV488 recombineering plasmid) (57), which had been thawed on ice. DNA was mixed with the cells and incubated on ice for 5 min and then electroporated at 1,600V. A volume of 250 µL THB +0.25 M sucrose was added immediately following electroporation, and the culture was incubated at 37°C for 2 h and then plated on THB agar +0.5 µg/mL erythromycin. Eight colonies were restreaked onto THB agar +0.5 µg/mL erythromycin, and colonies on those plates were used to inoculate 5 mL THB +0.5 µg/mL erythromycin +1 mM IPTG to remove the recombineering plasmid. Individual clones from those plates were streaked onto THB agar +0.5 µg/mL erythromycin and THB agar +2 µg/mL chloramphenicol to confirm loss of the plasmid. One colony that was positive for erythromycin resistance and negative for chloramphenicol resistance was used to create the frozen stock that was used for all subsequent studies. Whole-genome sequencing of *Spy∆fabM* was performed using the Bacterial Genome Sequencing service by Plasmidsaurus, Inc. This showed successful deletion of the *fabM* gene; however, there was an additional C -> T SNP in the genome at position 1,794,745, causing a premature stop codon in *gdpP,* a predicted cyclic-di-AMP-phosphodiesterase.

### S. pyogenes fabM-C

Three hundred base pairs upstream of the *S. pyogenes* 5448 *fabM* was amplified from 5448 gDNA using the primers spyfabM-upstream_fwd and spyfabM-upstream_rev. The 5448 *fabM* open reading frame and 76 bp downstream were amplified from 5448 using primers spyfabM-orf-plus76_fwd and spyfabM-orf-plus76_rev. A kanamycin resistance gene (kanR) was amplified from pSUGKBgl using primers spykanR-orf-fwd and spykanR-orf_rev. These three fragments were purified and extracted from an agarose gel and ligated together using Gibson Assembly Master Mix (NEB) according to the manufacturer’s instructions. The fully ligated product was amplified using primers spyfabM-upstream_fwd and spyfabM-orf-plus76_rev and purified and extracted from an agarose gel. This construct was used to create an *S. pyogenes* 5448 *fabM* complement strain using the pAV488 recombineering plasmid as described above for the *S. pyogenes ∆fabM* strain. Positive clones were selected by plating on THB agar +1 mg/mL kanamycin. Eight colonies were restreaked onto THB agar +1 mg/mL kanamycin, and colonies on those plates were used to inoculate 5 mL THB +1 mg/mL kanamycin +1 mM IPTG to remove the recombineering plasmid. Individual clones from those plates were streaked onto THB agar +1 mg/mL erythromycin and THB agar +2 µg/mL chloramphenicol to confirm loss of the plasmid. One colony that was positive for erythromycin resistance and negative for chloramphenicol resistance was used to create the frozen stock that was used for all subsequent studies. Whole-genome sequencing of *Spy-fabM-C* showed successful complementation of *fabM* but also a C -> T mutation at position 388,722, causing an S -> L mutation in the FabT transcriptional regulator, as well as a 281-bp insertion in the intergenic region of the rRNA/tRNA operon at position 553,154.

### S. pneumoniae ∆fabM

An *S. pneumoniae fabM* deletion mutant strain (*Spn∆fabM*) was constructed using a modified method based on a previously described protocol (58). Briefly, genomic DNA from *S. pneumoniae* TIGR4 (59) was extracted using the Quick-DNA Fungal/Bacterial Miniprep Kit (Zymo, Inc.) according to the manufacturer’s instructions. The upstream and downstream flanking regions of the *fabM* gene were PCR-amplified and fused with an *ermB* resistance cassette derived from the pDCerm plasmid (60) using primers TIGR4-fabM-Up-F, TIGR4-fabM-Up-R, TIGR4-fabM-ermB-F, TIGR4-fabM-ermB-R, TIGR4-fabM-Dw-F, and TIGR4-fabM-Dw-R. An isogenic *S. pneumoniae* TIGR4 strain was transformed with the resulting PCR product to generate the *Spn∆fabM* mutant using CSP-mediated natural competence (61). Colonies exhibiting erythromycin resistance were identified as *Spn∆fabM,* and proper integration of the *ermB* cassette was confirmed using primers fabM-KO-conf-F and fabM-KO-conf-R and the Bacterial Genome Sequencing service by Plasmidsaurus, Inc. Whole-genome sequencing of our isolate of TIGR4 and the *Spn∆fabM* strain indicated successful deletion of *fabM* and also an A -> C SNP at position 439,789, causing an Asn -> Thr mutation in the pilus tip adhesin RrgA.

### S. pneumoniae fabM-C

A complemented strain carrying a *fabM*-expressing plasmid (*fabM*-C) was constructed using a modified method based on a previously described protocol (58). To construct the *fabM*-expressing plasmid, the *fabM* gene was PCR-amplified with EcoRI and BamHI restriction sites added at the 5′ and 3′ ends, respectively, using primers TIGR4-fabM-comp-F, TIGR4-fabM-comp-R, pDC123_universal_F, and pDC123_universal_R and ligated into the pDC123 plasmid (62), which carries a chloramphenicol resistance cassette (63). The *Spn∆fabM* strain was transformed with the *fabM*-expressing plasmid using CSP-mediated natural competence (61) to generate the complemented strain. Colonies exhibiting resistance to both erythromycin and chloramphenicol were identified as fabM-C. Whole-genome sequencing of *Spn-fabM-C* showed successful complementation of *fabM,* as well as the SNP in *rrgA,* and also an additional G -> A SNP, which did not appear in the parent *Spn*∆*fabM* strain, at position 269,092, causing an M -> I mutation in folylpolyglutamate synthase.

### *S. mutans* p*comDE-*luc and *S. mutans* p*comDE-*luc-∆*fabM* reporter strains

Using overlapping primers pcomDE-R, pcomDE-R, DERluc-F, DERluc-R, T1F5, T1F3, Tuf5, KT13, T1R5, and T1R3, a construct was made using PCR and Gibson Assembly containing 461 bp upstream of *comE* (i.e., the *comCDE* promoter), followed by the open reading frame of Renilla luciferase (*Rluc*), followed by the constitutively active *S. mutans tuf* promoter, followed by a kanamycin resistance gene *kanR,* flanked by 500 bp regions upstream and downstream of the SMU.1180c gene, such that the construct would insert into the SMU.1180c gene. SMU.1180c was chosen as an integration site based on previously described *S. mutans* expression vectors (64). This construct was transformed into *S. mutans* UA159 or *Smu∆fabM* using CSP-induced transformation. Briefly, an overnight culture of *S. mutans* was diluted 1:50 with fresh THB. An aliquot of 200 μL of the diluted culture was transferred to a sterile well of a 96-well plate and mixed with CSP (500 nM) and target DNA. After incubation at 37°C with 5% CO_2_ for another 2 h, the culture was streaked on THB plates supplemented with the appropriate antibiotic concentration. Colony formation on was assessed after 2 days and one colony with expected resistance profile had the proper mutations confirmed by PCR and was used for downstream experiments.

### S*. mutans pnlmA-mCherry* and *S. mutans pnlmA-mCherry-∆fabM* reporter strains

Using overlapping primers P150-5, P150-3, T2nlmAcherryO5, T2nlmAmcherryO3, N2F5, N2F3, N2R5, and N2R3, a construct was made using PCR and Gibson Assembly containing 212 bp upstream of *nlmA* (i.e., the *nlmA* promoter), followed by the open reading frame of mCherry, followed by the *S. mutans tuf* promoter, followed by a kanamycin resistance gene *kanR,* flanked by 500-bp regions upstream and downstream of the SMU.221c gene, such that the construct would insert into the SMU.221c gene. SMU.221c was chosen as an insertion site since this gene is annotated as a site-specific integrase with no adjacent gene, therefore likely representing a phage relic where any polar effects would be minimal. This construct was transformed into *S. mutans* UA159 or *Smu∆fabM* using CSP-induced transformation. Briefly, an overnight culture of *S. mutans* was diluted 1:50 with fresh THB. An aliquot of 200 μL of the diluted culture was transferred to a sterile well of a 96-well plate and mixed with CSP (500 nM) and target DNA. After incubation at 37°C with 5% CO_2_ for another 2 h, the culture was streaked on THB plates supplemented with the appropriate antibiotic concentration. Colony formation was assessed after 2 days and one colony with expected resistance profile had the proper mutations confirmed by PCR and was used for downstream experiments.

### Growth curves

Overnight cultures of the indicated strains were normalized to the OD_600_ of the lowest density culture in the comparison using the indicated growth media. Growth curves were performed in either 96-well or 384-well clear plates (Corning, Inc.). For growth curves in 96-well plates, 10 µL of the overnight cultures was added to 200 μL of the indicated growth media with or without indicated compounds. For growth curves using 384-well plates, 2.5 µL of the overnight cultures was added to 50 µL of the indicated growth media with or without the indicated compounds. Plates were sealed with the Breathe-Easy sealing membrane (Diversified Biotech), and growth curves were performed without the plastic plate lid. Growth was monitored using an Infinite Nano (Tecan Group, Ltd.). Optical density at 600 nm (OD_600_) (and mCherry fluorescence or luciferase luminescence, when applicable) was measured every 30 min for the indicated amount of time under 37 °C, with 5 s of shaking prior to each reading. Four to eight replicates of each strain were monitored.

### Derivatization of fatty acid methyl esters for GC-MS

Cultures of the indicated strains and growth media were incubated at 37°C in a 5% (vol/vol) CO_2_–95% air environment for the indicated amount of time. Cells were harvested by centrifugation at 4,000 × *g* for 10 min. The resulting pellets were decanted and stored at −80°C. Pellets were resuspended and then vortexed in 100 µL concentrated sulfuric acid (96%) and 200 µL methanol. Samples were then boiled by incubation in boiling water for 5 min and allowed to cool to RT. Three hundred microliters of dichloromethane was added, and samples were then vortexed and centrifuged for 1 min at 15,000 × *g*. The organic (bottom) layer was transferred to a new tube containing a pinch of Na_2_SO_4_, mixed by vortexing, and centrifuged for 1 min at 15,000 × *g*. The supernatant was then transferred to an HPLC tube and stored at 4°C until being loaded on the GC-MS.

### GC-MS

One microliter of the sample was injected into a Shimadzu GC-2010 GC-MS equipped with an AV-1701 capillary GC column (Zebron, Inc.) using an AOC20i auto injector (Shimadzu). The carrier gas was helium, and the column oven temperature program was as follows: 45°C for 2 min; ramp temperature 9°/min to 180°C and then hold for 5 min; ramp temperature 40°/min to 220°C and then hold for 5 min; ramp temperature 40°/min to 240°C and then hold for 11.5 min; ramp temperature 40°/min to 280°C and hold for 2 min (43 min total). Spectra from 20 to 650 *m/z* were collected from 6 min to 37.5 min with a scan speed of 10,000 and an event time of 0.1 s. The ion source temperature was 200°C, and the interface temperature was 250°C. .qgd data files were converted to CDF files using GCMS Solutions Postrun Analysis (Shimadzu). CDF files were converted to .mzML files using MZmine (65). Spectral alignment, deconvolution, integration, as well as library search were performed using MS-Hub as part of the Global Natural Products Social Molecular Networking (GNPS) GC-MS EI Data Analysis pipeline (66). Identity of peaks was also confirmed based on the comparison of retention times and mass spectra to a known standard component of TraceCERT 37 Component FAME Mix (Supelco).

### Deferred antagonism (i.e., “competition”) assay

The deferred antagonism assay was performed as previously described (67). Briefly, 8 mL of overnight cultures of the initial colonizer was spotted onto THB 1% agar and incubated overnight at 37°C under 5% CO2/95% air. The following day, the plates were sterilized using the sterilization setting (90 s) in a GS Gene Linker UV Chamber (Bio-Rad, Inc.). Five hundred microliters of overnight cultures of *S. sanguinis* SK36, *S. gordonii* ATCC 10558, or *S. mitis* F0392 was added to 5 mL molten THB 0.75% agar that had been cooled to 40°C, and this was used to overlay the plates with the *S. mutans* colonies. The agar overlay was allowed to solidify at room temperature, and then the plates were incubated overnight at 37°C under 5% CO2/95% air. Zones of inhibition were examined and photographed the following day.

### Scanning electron microscopy

Overnight cultures of the indicated strains were used to inoculate 1 mL of THB in a 24-well plate (Corning, Inc.) containing a Thermanox disk (Nunc). Plates were incubated overnight at 37°C in a 5% (vol/vol) CO2–95% air environment. The following day, the medium was gently aspirated from the wells, and 1 mL fixing agent (2.5% formaldehyde/glutaraldehyde in 0.1 M sodium cacodylate buffer pH 7.4 [Electron Microscopy Sciences]) was added. Cells were fixed for at least 1 h at 4°C. Samples were dehydrated using an ethanol gradient and then a critical point dryer (Leica CPD300) prior to sputter coating with 10-nm-thick carbon (ACE600). Cells were imaged on an Apreo scanning electron microscope (Thermo Fisher Scientific). Sample preparation beyond the fixing and imaging were performed at the OHSU Multiscale Microscopy Core, a member of the OHSU University Shared Resource Cores. RRID:SCR_009969.

### Neutrophil killing assay

#### Neutrophil preparation

Blood sample collection protocols were reviewed by the Oregon Health and Science University Institutional Review Board (IRB) prior to the initiation of the study and deemed to be not human subject research (#20759). Venous blood was collected at the Oregon Clinical and Translational Research Institute (OCTRI) from healthy donors, and neutrophils were isolated as previously described (68). Briefly, red blood cells were removed by dextran sedimentation followed by Ficoll gradient centrifugation and hypotonic lysis. Neutrophils were subsequently resuspended in phosphate-buffered saline (without calcium and magnesium) containing 0.1% dextrose and used immediately.

#### Serum preparation

Blood was drawn from a healthy donor as described above and added to a serum tube. The serum tube containing the blood was placed on a rocker for 30 min at RT. The serum was separated from the resulting clot by centrifugation at 1,000 × *g* for 10 min. The serum was then aliquoted into two equal volumes in 15-mL centrifuge tubes. One tube was heat-inactivated in a 56°C water bath for 30 min.

#### Neutrophil killing assay

Mid-log phase bacterial cells of the indicated strain were harvested by centrifugation at 4,000 × *g* for 10 min. The pellet was decanted and subsequently resuspended in HBSS+/+ buffer such that an equal 10 µL volume of bacterial cells would have the desired MOI compared to a 10 µL volume of neutrophils. Ten microliters of bacteria in HBSS+/+ was added to a round bottom 96-well plate (Corning, Inc.). Five microliters of serum was added to the bacteria and incubated for 30 min on a rocker at RT. Ten microliters of neutrophils or 10 µl of HBSS+/+ were then added, and the total volume was brought to 100 µL by addition of 75 µL of HBSS+/+. The plate was centrifuged at 500 × *g* for 5 min. After the indicated amount of time incubating at 37°C, the samples were added to 100 µL of ice-cold 0.3% saponin and incubated on ice for 2 min. The samples were then serially diluted in DMEM out to 1 × 10^−8^, plated on THB agar, and incubated overnight at 37°C in a 5% (vol/vol) CO_2_–95% air environment.

## Data Availability

The genome sequences and raw sequencing reads for the whole-genome sequencing of *Spy∆fabM, SpyfabM-C, Spn∆fabM,* and *Spn-fabM-C* are available on NCBI with the accession number PRJNA1474126.

## SUPPLEMENTAL MATERIAL

Figure S1 jb.00026-26-s0001.pdf Phenotypes of the *S. pyogenes* and *S. pneumoniae* ∆*fabM* complement strains.

Figure S2 jb.00026-26-s0002.pdf Exogenous unsaturated fatty acids can rescue growth of ∆*fabM* strains.

Figure S3 jb.00026-26-s0003.pdf ∆*fabM* strains have decreased resistance to environmental stress.

Figure S4 jb.00026-26-s0004.pdf Complementation of fabM rescues inhibition of *S. sanguinis* by *S. mutans*.

Figure S5 jb.00026-26-s0005.pdf Impact of fabM deletion or fatty acid supplementation on cell chain length.

Supplemental legends jb.00026-26-s0006.docx Legends for Fig. S1 to S5.

Table S1 jb.00026-26-s0007.xlsx Strains, plasmids, and primers used in this study.

ASM does not own the copyrights to Supplemental Material that may be linked to, or accessed through, an article. The authors have granted ASM a non-exclusive, world-wide license to publish the Supplemental Material files. Please contact the corresponding author directly for reuse.
