# Supplementary material for: Monounsaturated fatty acid biosynthesis is critical for streptococcal envelope homeostasis and stress tolerance
Source: J Bacteriol. 2026 Aug 7;208(9):e00026-26. doi: 10.1128/jb.00026-26 (PMC13584702; doi:10.1128/jb.00026-26)
Supplement: Supplementary file 1 [file jb.00026-26-s0001.pdf]

Figure S1

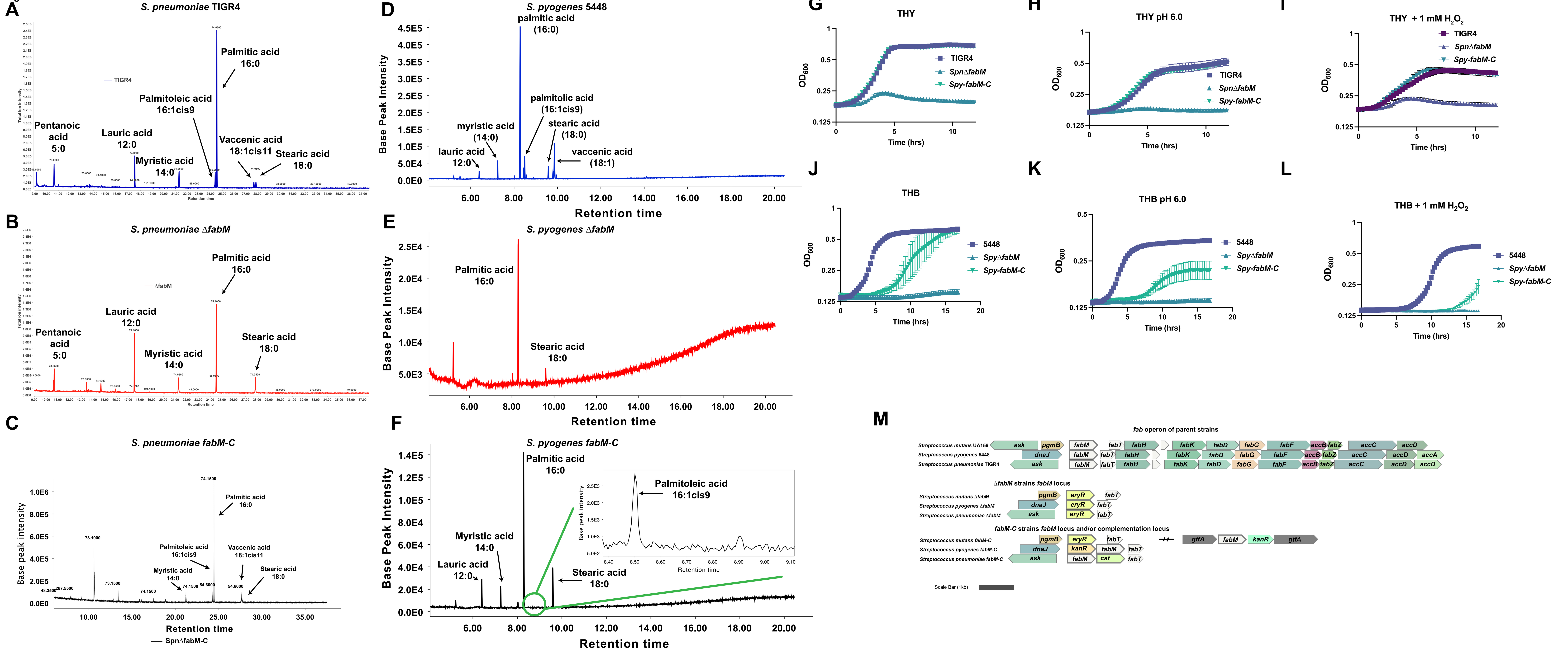

A
S. pneumoniae TIGR4
TIGR4
Pentanoic acid 5:0
Lauric acid 12:0
Myristic acid 14:0
Palmitoleic acid 16:1cis9
Palmitic acid 16:0
Vaccenic acid 18:1cis11
Stearic acid 18:0
B
S. pneumoniae ΔfabM
ΔfabM
Pentanoic acid 5:0
Lauric acid 12:0
Myristic acid 14:0
Palmitic acid 16:0
Stearic acid 18:0
C
S. pneumoniae fabM-C
Base peak intensity
Palmitic acid 16:0
Palmitoleic acid 16:1cis9
Vaccenic acid 18:1cis11
Myristic acid 14:0
Stearic acid 18:0
Retention time
SpnΔfabM-C
D
S. pyogenes 5448
palmitic acid (16:0)
palmitolic acid (16:1cis9)
stearic acid (18:0)
myristic acid (14:0)
lauric acid 12:0
vaccenic acid (18:1)
Base Peak Intensity
Retention time
E
S. pyogenes ΔfabM
Palmitic acid 16:0
Stearic acid 18:0
Base Peak Intensity
Retention time
F
S. pyogenes fabM-C
Palmitic acid 16:0
Palmitoleic acid 16:1cis9
Myristic acid 14:0
Lauric acid 12:0
Stearic acid 18:0
Base peak intensity
Retention time
Base Peak Intensity
Retention time
G
THY
TIGR4
SpnΔfabM
Spy-fabM-C
OD600
Time (hrs)
H
THY pH 6.0
TIGR4
SpnΔfabM
Spy-fabM-C
I
THY + 1 mM H2O2
TIGR4
SpnΔfabM
Spy-fabM-C
J
THB
5448
SpyΔfabM
Spy-fabM-C
K
THB pH 6.0
5448
SpyΔfabM
Spy-fabM-C
L
THB + 1 mM H2O2
5448
SpyΔfabM
Spy-fabM-C
M
fab operon of parent strains
Streptococcus mutans UA159
Streptococcus pyogenes 5448
Streptococcus pneumoniae TIGR4
ask
pgmB
dnaJ
fabM
fabT
fabH
fabK
fabD
fabG
fabF
accB
fabZ
accC
accD
accA
ΔfabM strains fabM locus
Streptococcus mutans ΔfabM
Streptococcus pyogenes ΔfabM
Streptococcus pneumoniae ΔfabM
eryR
fabM-C strains fabM locus and/or complementation locus
Streptococcus mutans fabM-C
Streptococcus pyogenes fabM-C
Streptococcus pneumoniae fabM-C
kanR
cat
gtfA
Scale Bar (1kb)
