# Supplementary material for: Monounsaturated fatty acid biosynthesis is critical for streptococcal envelope homeostasis and stress tolerance
Source: J Bacteriol. 2026 Aug 7;208(9):e00026-26. doi: 10.1128/jb.00026-26 (PMC13584702; doi:10.1128/jb.00026-26)

Figure S2

Exogenous unsaturated fatty acids can rescue growth of Δ*fabM* strains

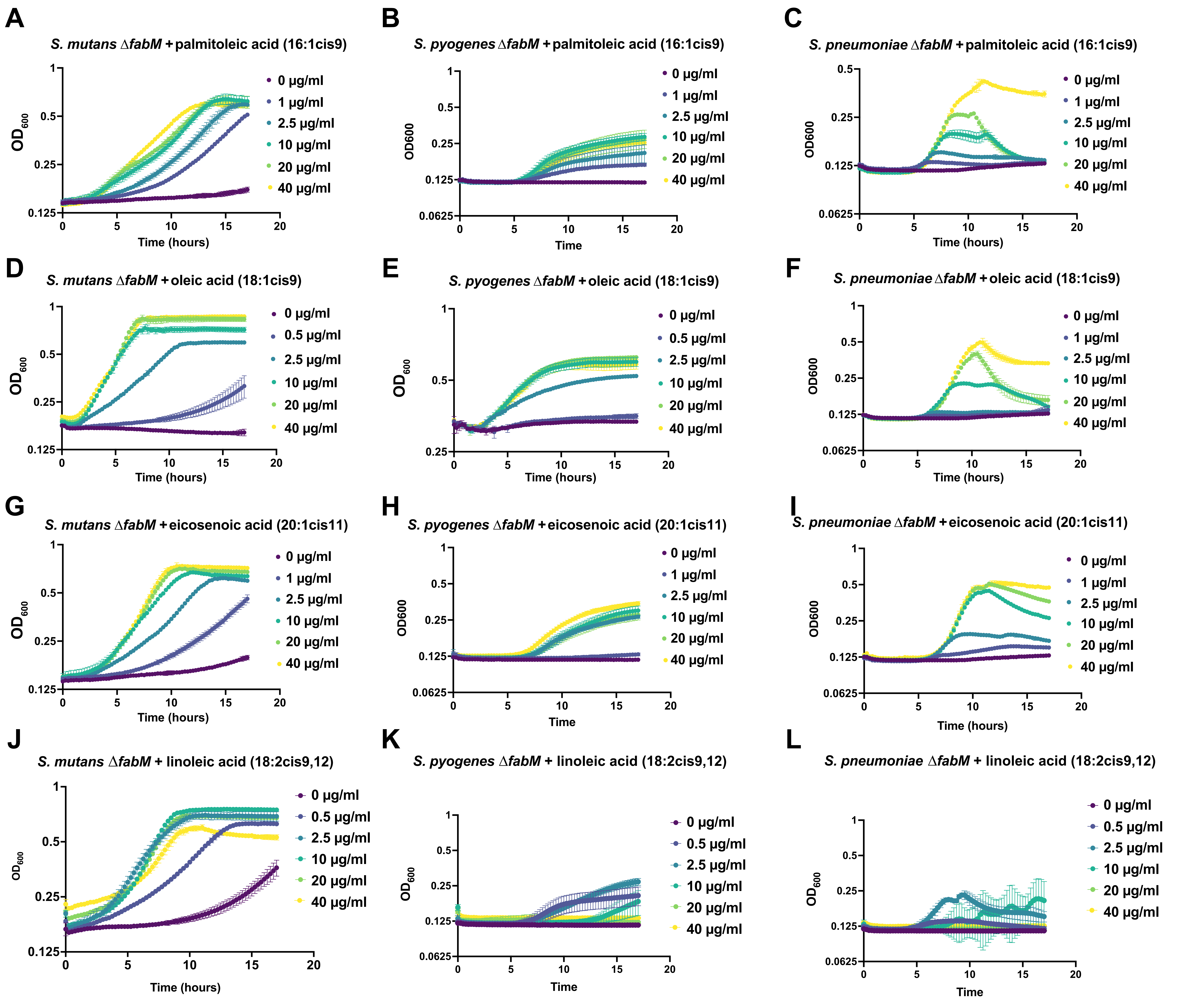

Supplement: Supplementary file 2 [file jb.00026-26-s0002.pdf]
