# Supplementary material for: Monounsaturated fatty acid biosynthesis is critical for streptococcal envelope homeostasis and stress tolerance
Source: J Bacteriol. 2026 Aug 7;208(9):e00026-26. doi: 10.1128/jb.00026-26 (PMC13584702; doi:10.1128/jb.00026-26)

Figure S3

ΔfabM strains have decreased resistance to environmental stress

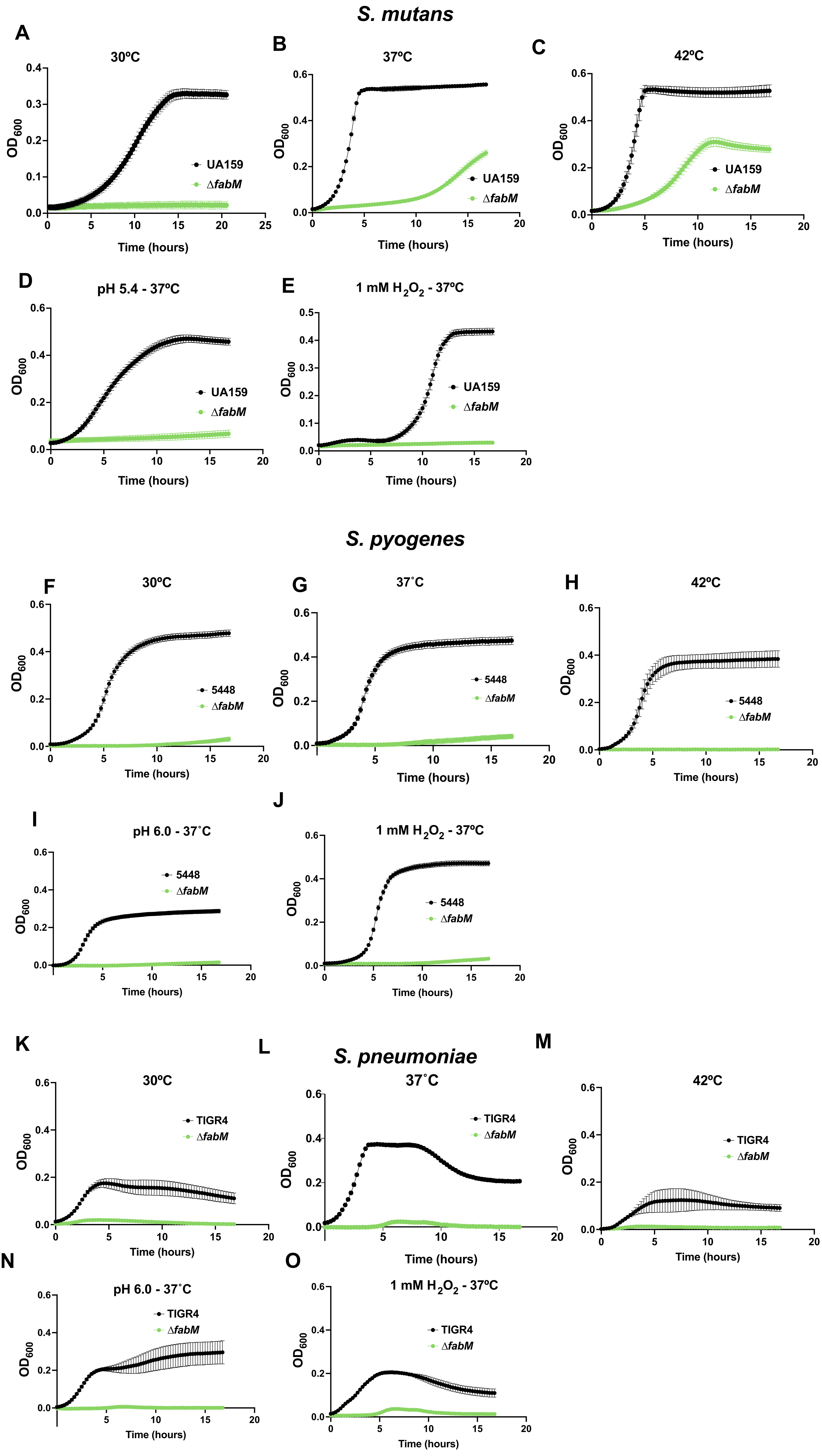

Supplement: Supplementary file 3 [file jb.00026-26-s0003.pdf]
