# Supplementary material for: Monounsaturated fatty acid biosynthesis is critical for streptococcal envelope homeostasis and stress tolerance
Source: J Bacteriol. 2026 Aug 7;208(9):e00026-26. doi: 10.1128/jb.00026-26 (PMC13584702; doi:10.1128/jb.00026-26)
Supplement: Supplementary file 4 [file jb.00026-26-s0004.pdf]

Figure S4

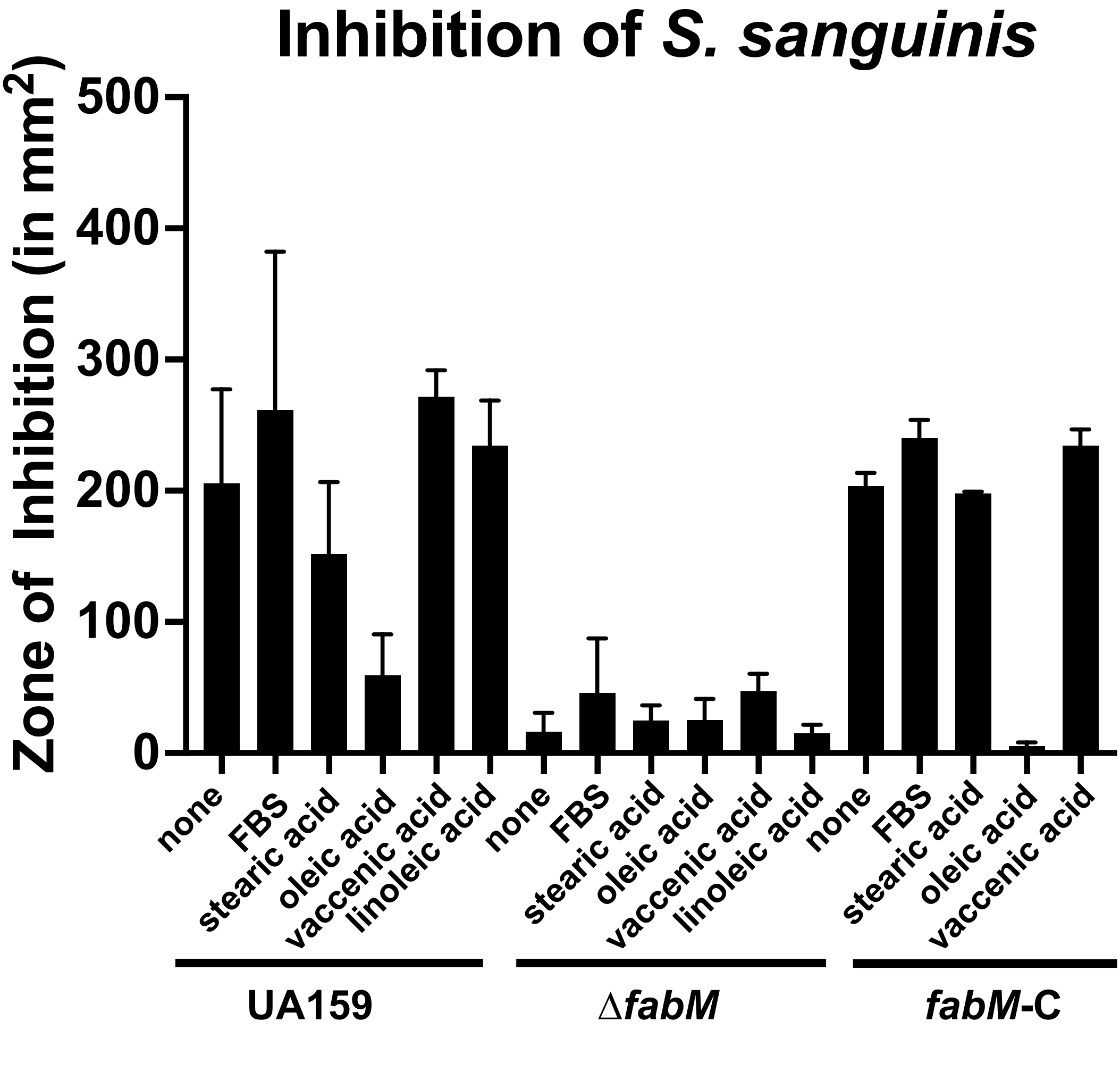
Inhibition of S. sanguinis
Zone of Inhibition (in mm2)
500
400
300
200
100
0
none
FBS
stearic acid
oleic acid
vaccenic acid
linoleic acid
none
FBS
stearic acid
oleic acid
vaccenic acid
linoleic acid
none
FBS
stearic acid
oleic acid
vaccenic acid
UA159
ΔfabM
fabM-C
