# Supplementary material for: Monounsaturated fatty acid biosynthesis is critical for streptococcal envelope homeostasis and stress tolerance
Source: J Bacteriol. 2026 Aug 7;208(9):e00026-26. doi: 10.1128/jb.00026-26 (PMC13584702; doi:10.1128/jb.00026-26)
Supplement: Supplementary file 5 [file jb.00026-26-s0005.pdf]

Figure S5

A.

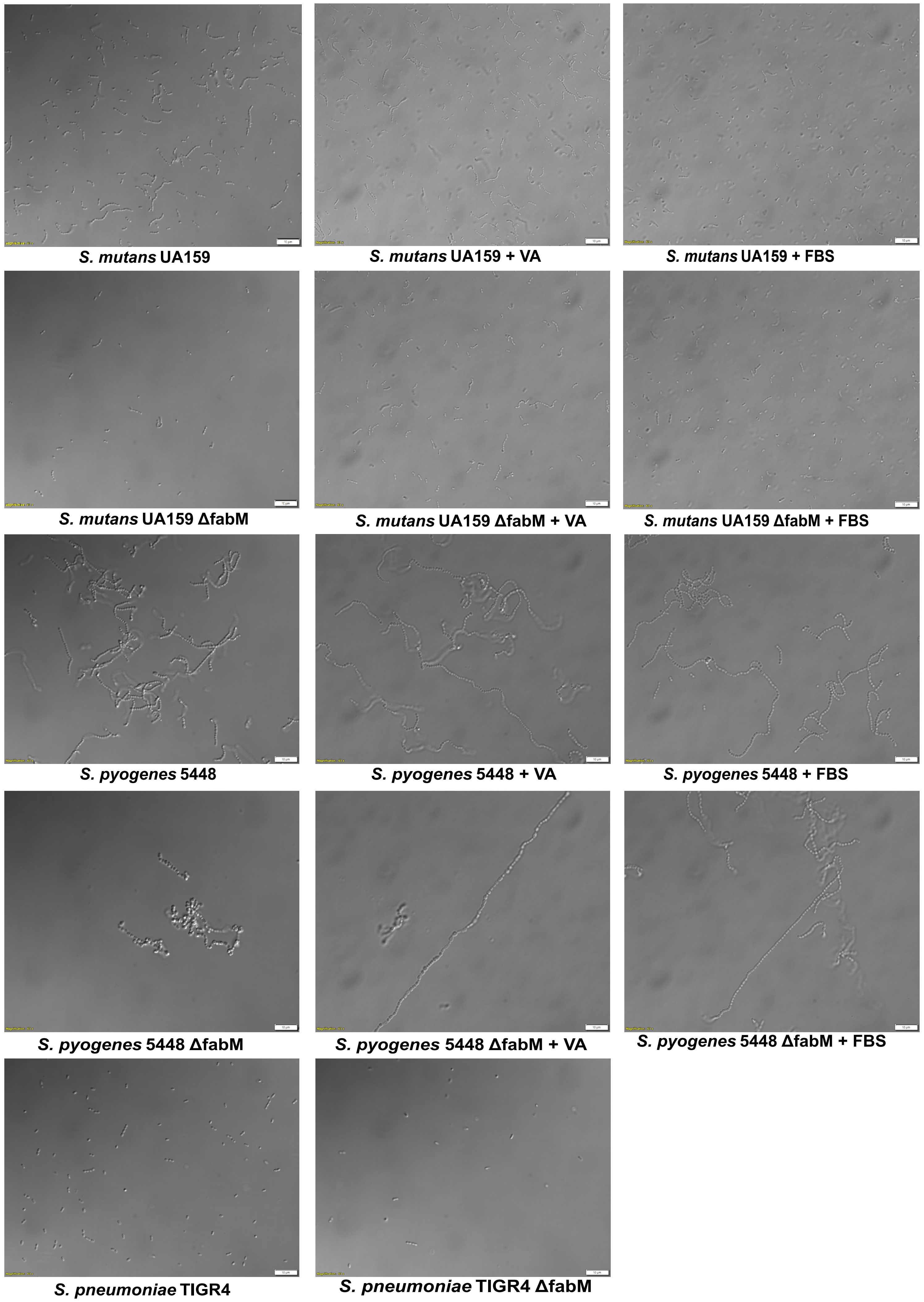
S. mutans UA159
S. mutans UA159 + VA
S. mutans UA159 + FBS
S. mutans UA159 ΔfabM
S. mutans UA159 ΔfabM + VA
S. mutans UA159 ΔfabM + FBS
S. pyogenes 5448
S. pyogenes 5448 + VA
S. pyogenes 5448 + FBS
S. pyogenes 5448 ΔfabM
S. pyogenes 5448 ΔfabM + VA
S. pyogenes 5448 ΔfabM + FBS
S. pneumoniae TIGR4
S. pneumoniae TIGR4 ΔfabM

B.

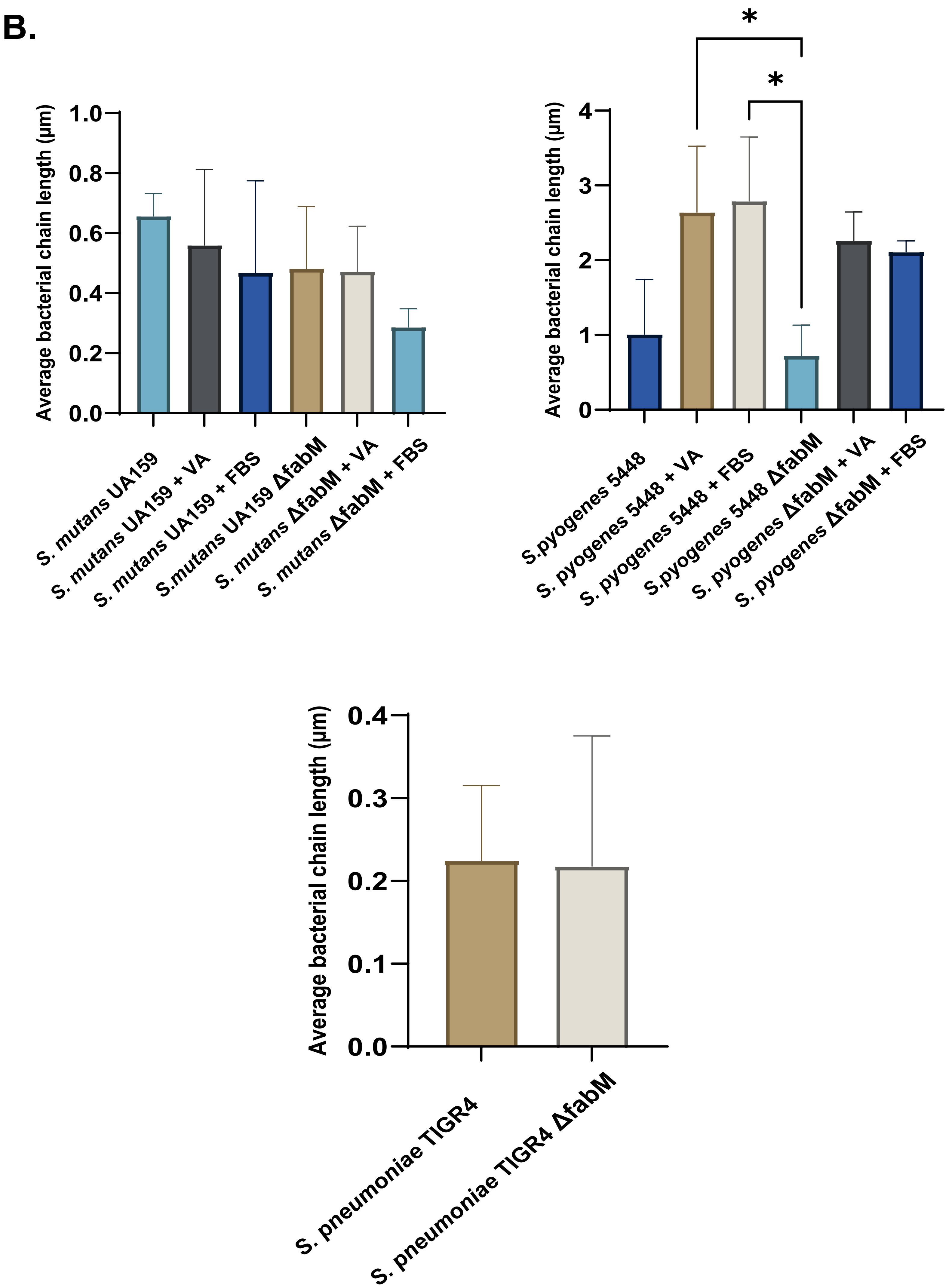
Average bacterial chain length (µm)
1.0
0.8
0.6
0.4
0.2
0.0
S. mutans UA159
S. mutans UA159 + VA
S. mutans UA159 + FBS
S.mutans UA159 ΔfabM
S. mutans ΔfabM + VA
S. mutans ΔfabM + FBS
*
*
Average bacterial chain length (µm)
4
3
2
1
0
S.pyogenes 5448
S. pyogenes 5448 + VA
S. pyogenes 5448 + FBS
S.pyogenes 5448 ΔfabM
S. pyogenes ΔfabM + VA
S. pyogenes ΔfabM + FBS
Average bacterial chain length (µm)
0.4
0.3
0.2
0.1
0.0
S. pneumoniae TIGR4
S. pneumoniae TIGR4 ΔfabM
