# Supplementary material for: Monounsaturated fatty acid biosynthesis is critical for streptococcal envelope homeostasis and stress tolerance
Source: J Bacteriol. 2026 Aug 7;208(9):e00026-26. doi: 10.1128/jb.00026-26 (PMC13584702; doi:10.1128/jb.00026-26)
Supplement: Supplementary file 6 [file jb.00026-26-s0006.docx]

**Supplemental Figure Legends**

**Figure S1: Phenotypes of the *S. pyogenes* and *S. pneumoniae ∆fabM* complement strains.** Panels A-F, Chromatograms from GC-MS of the indicated strain. Peaks are labeled with the mass to charge ratio (m/z; which is approximately 74 for SFAs and 55 for MUFAs), common name, and lipid number. Peaks were identified based on having the same retention time and mass spectra as known standards. The inset for *Spy fabM-C* is a zoom of the indicated retention time to show the peak for the MUFA palmitoleic acid. Panel G-L growth curves illustrating rescue of growth by the *S. pyogenes* and *S. pneumoniae ∆fabM* complement strains in the indicated conditions. Panel M, illustration showing the layout of the *fab* operon across *S. mutans, S. pyogenes,* and *S. pneumoniae,* as well as the *fabM* loci in the indicated *∆fabM* and *fabM* complement strains.

**Figure S2: Exogenous unsaturated fatty acids can rescue growth of ∆*fabM* strains.** Expanded set of growth curves from Figure 2, showing all tested strains and fatty acids.

**Figure S3: ∆*fabM* strains have decreased resistance to environmental stress.** Expanded set of growth curves from Figure 3, showing all tested strains and conditions.

**Figure S4: Complementation of *fabM* rescues inhibition of *S. sanguinis* by *S. mutans.*** Expanded panel from Figure 4A including the *Smu-fabM-C* strain.

**Figure S5: Impact of *fabM* deletion or fatty acid supplementation on cell chain length.** Panel A shows representative images taken by light microscopy under 63X magnification of the indicated strain and growth media. Panel B is bar graphs illustrating the quantification of the chain lengths under the conditions shown in Panel A. *denotes statistical significance (p < 0.05) between indicated groups of samples based on a Tukey’s Honestly Significant Differences test following a one-way ANOVA.
